# Diverse Intrinsic Properties Shape Functional Phenotype of Low-Frequency Neurons in the Auditory Brainstem

**DOI:** 10.3389/fncel.2018.00175

**Published:** 2018-06-26

**Authors:** Hui Hong, Xiaoyu Wang, Ting Lu, Diego A. R. Zorio, Yuan Wang, Jason Tait Sanchez

**Affiliations:** ^1^Roxelyn and Richard Pepper Department of Communication Sciences and Disorders, Northwestern University, Evanston, IL, United States; ^2^Department of Biomedical Sciences, Florida State University, Tallahassee, FL, United States; ^3^Program in Neuroscience Florida State University College of Medicine, Florida State University, Tallahassee, FL, United States; ^4^Department of Neurobiology, Northwestern University, Evanston, IL, United States; ^5^The Hugh Knowles Hearing Research Center, Northwestern University, Evanston, IL, United States

**Keywords:** nucleus magnocellularis, potassium channels, sodium channels, action potentials, auditory brainstem, resurgent sodium current, tonotopic map

## Abstract

In the auditory system, tonotopy is the spatial arrangement of where sounds of different frequencies are processed. Defined by the organization of neurons and their inputs, tonotopy emphasizes distinctions in neuronal structure and function across topographic gradients and is a common feature shared among vertebrates. In this study we characterized action potential firing patterns and ion channel properties from neurons located in the extremely low-frequency region of the chicken nucleus magnocellularis (NM), an auditory brainstem structure. We found that NM neurons responsible for encoding the lowest sound frequencies (termed NMc neurons) have enhanced excitability and fired bursts of action potentials to sinusoidal inputs ≤10 Hz; a distinct firing pattern compared to higher-frequency neurons. This response property was due to lower amounts of voltage dependent potassium (K_V_) conductances, unique combination of K_V_ subunits and specialized sodium (Na_V_) channel properties. Particularly, NMc neurons had significantly lower K_V_1 and K_V_3 currents, but higher K_V_2 current. NMc neurons also showed larger and faster transient Na_V_ current (*I*_NaT_) with different voltage dependence of inactivation from higher-frequency neurons. In contrast, significantly smaller resurgent sodium current (*I*_NaR_) was present in NMc with kinetics and voltage dependence that differed from higher-frequency neurons. Immunohistochemistry showed expression of Na_V_1.6 channel subtypes across the tonotopic axis. However, various immunoreactive patterns were observed between regions, likely underlying some tonotopic differences in *I*_NaT_ and *I*_NaR_. Finally, using pharmacology and computational modeling, we concluded that K_V_3, K_V_2 channels and *I*_NaR_ work synergistically to regulate burst firing in NMc.

## Introduction

Topography in the vertebrate brain represents an orderly organization of neuronal architecture responsible for encoding sensory information. Topography in the auditory system is defined by tonotopy – the spatial arrangement of structures that subserve the processing of different sound frequencies. Tonotopy originates along the peripheral sensory epithelium and is preserved throughout the auditory system. Tonotopy in the central pathway is arranged not only by specific location of neurons and their inputs, but by differences in their structural and functional properties along the tonotopic axis (Koppl, 1994; Koppl and Carr, 1997; Brew and Forsythe, 2005; Wang et al., 2017). An example is the tonotopic arrangement of the avian nucleus magnocellularis (NM); the analog of the mammalian anteroventral cochlear nucleus. NM neurons are distributed from caudolateral to rostromedial, with neurons encoding the lowest sound frequencies for chickens located at the extreme caudolateral pole, referred here as NMc neurons (Rubel and Parks, 1975; Wang et al., 2017). Although the hearing range and sensitivity of many species of birds have been known for decades (Sachs et al., 1978), the majority of studies have focused on mid- to high-frequency regions. Few exceptions are the homing pigeon and the domesticated chicken, which can hear sounds as low as 2 Hz (i.e., infrasound) and as high as 9 kHz (Kreithen and Quine, 1979; Hill et al., 2014). However, as both studies noted, the birds’ perception of infrasound was unique compared to higher-frequency sounds, suggesting alternative mechanisms for encoding extremely low frequencies < 20 Hz (Schermuly and Klinke, 1990).

**FIGURE 1:**
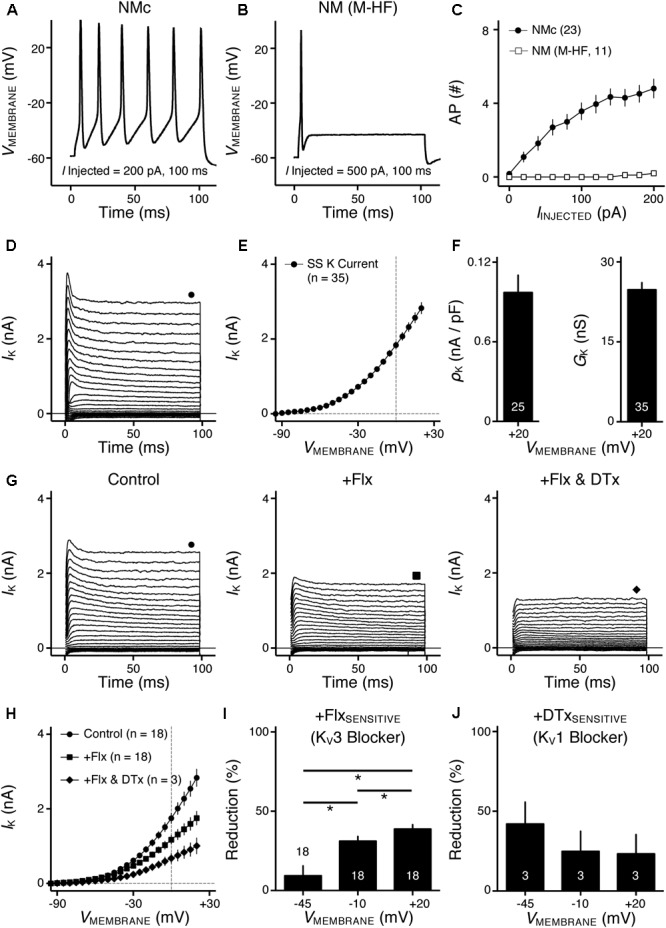
Voltage dependent potassium (K_V_) current properties of NMc neurons. **(A)** Representative membrane response recorded from an NMc neuron to current injection of 200 pA for 100 ms. **(B)** Representative membrane response recorded from a mid- to high-frequency (M-HF) NM neuron to current injection of 500 pA for 100 ms. **(C)** Average population data showing differences in the number of APs as a function of current injection for NMc and mid- to high-frequency (M-HF) NM neurons. Data in **(C)** were modified from our previous study (see Wang et al., 2017; **Figure 10D**). **(D)** Representative K_V_ current traces (*I*_K_) recorded from an NMc neuron, in response to membrane voltages clamped from –100 to +20 mV (Δ step = 5 mV, duration = 100 ms). The holding voltage is –70 mV. Steady-state (SS) K_V_ currents were measured at the end of current traces (filled circle). **(E)** Population data showing the relationship of steady-state (SS) K_V_ currents to membrane voltages (*V*_MEMBRANE_). Note that data point at –100 mV is not shown for simplicity. **(F)** Population data showing the K_V_ current density (ρ_K_) and conductance (*G*_K_) at the membrane voltage (*V*_MEMBRANE_) of +20 mV. Numbers in bars represent sample size. **(G)** Representative K_V_ current traces (*I*_K_) in control and during drug application. Flx, fluoxetine (100 μM). DTx, dendrotoxin (0.1 μM). Symbols (circle, square, and diamond) at the end of current traces represent time window of measured steady-state (SS) K_V_ currents. **(H)** Population data showing the relationship of steady-state (SS) K_V_ currents to membrane voltages (*V*_MEMBRANE_) in control and during drug application. **(I,J)** Population data showing the percent reduction in steady-state (SS) K_V_ currents due to the application of Flx **(I)** or DTx **(J)**, at membrane voltages (*V*_MEMBRANE_) of +20, –10 and –45 mV. Error bar = standard error.

Indeed, we previously reported that low-frequency NMc neurons differ notably from higher-frequency NM neurons in several ways (Wang et al., 2017). First, NMc neurons have elaborate dendritic processes and form bouton synapses with auditory nerve inputs. Based on their heterogeneous dendritic structures, NMc neurons are further divided into two subtypes, denoted as NMc1 and NMc2. In contrast, mid- to high-frequency NM neurons located more rostrally are relatively homogenous, adendritic and receive input from a few auditory nerve fibers through large endbulb of Held synapses (Jhaveri and Morest, 1982a,b). Second, NMc neurons show enhanced neuronal excitability and distinct action potential (AP) firing patterns compared to their higher-frequency counterparts. NMc neurons are spontaneously active *in vitro* and able to fire repetitive APs in response to small amount of sustained current injections (i.e., low threshold current, **Figures 1A,C**) (Wang et al., 2017). Mid- to high-frequency NM neurons do not fire spontaneously *in vitro*, require higher threshold current and only generate a single onset AP in response to sustained current injections (**Figures 1B,C**) (Hong et al., 2016). NMc neurons also show significantly longer time constant and larger input resistance. Based on these aforementioned discrepancies across tonotopic regions, we hypothesized that NMc neurons have distinct voltage dependent potassium (K_V_) and sodium (Na_V_) channel properties, which play an important role in shaping specific physiological phenotype of NMc neurons.

This hypothesis is partially supported by lower levels of K_V_ channel immunoreactivity in the low-frequency, caudolateral region of NM (Parameshwaran et al., 2001; Fukui and Ohmori, 2004). Kuba and Ohmori (2009) also showed larger Na_V_ current amplitude and higher Na_V_ channel density, as indicated by stronger immunoreactivity, toward lower-frequency NM. Furthermore, we showed that Na_V_1.6-channel subtype is expressed in mid- to high-frequency NM and carry robust resurgent Na_V_ current (*I*_NaR_) (Hong et al., 2017). *I*_NaR_ is a prevalent property conserved in auditory structures of both avians and mammals and plays an important role in high-frequency AP firing of peripheral and brainstem neurons (Leao K.E. et al., 2006; Kim et al., 2010; Browne et al., 2017; Hong et al., 2017). It remains to be determined if NMc neurons have different Na_V_ channel properties compared to mid- to high-frequency neurons and to what extent – if any – *I*_NaR_ present with tonotopic heterogeneity that contributes to the distinct AP firing pattern of NMc neurons.

The current study addresses these issues by characterizing the underlying K_V_ and Na_V_ channel properties that contribute to the functional phenotype of NMc neurons and is a follow-up to our first report regarding these neurons (Wang et al., 2017). To ensure fair comparisons across tonotopic regions of NM, we applied similar methods of electrophysiology, immunocytochemistry and computational modeling we previously reported (Hong et al., 2016, 2017). We found that NMc neurons burst fired at relatively fast rates to low-frequency sinusoidal current injections that was partially attributed to reduced K_V_1 and K_V_3 currents but higher K_V_2 currents – properties that differ from higher-frequency neurons (Hong et al., 2016). NMc neurons also presented with specialized Na_V_ channel properties, including *I*_NaR_. The presence of *I*_NaR_ increased availability of Na_V_ channels and facilitated their recovery shortly after depolarization. Removal of *I*_NaR_ in our model NMc neuron reduced its capability to burst fire. Immunocytochemistry confirmed the expression of K_V_3.1, K_V_2.2, and Na_V_1.6 in NMc and demonstrated their distinct distribution patterns. The synergy of K_V_3-, K_V_2-containing channels and *I*_NaR_ help shape the functional phenotype of NMc neurons and highlights the significant biological variation of the auditory brainstem in processing sound information of varying frequencies.

## Materials and Methods

### Ethical Approval

All animal procedures were approved by the Northwestern University and Florida State University Institutional Animal Care and Use Committees and conducted in accordance with the National Institutes of Health Guide for the Care and Use of Laboratory Animals. For electrophysiological experiments, acute brainstem slices were prepared from White Leghorn chicken (*Gallus gallus domesticus*) embryos of either sex as previously described (Sanchez et al., 2011; Hong et al., 2016). Briefly, embryos were rapidly decapitated and the brain was dissected from the skull to isolate the brainstem region of interest. This procedure is consistent with the recommendation from the Panel on Euthanasia of the American Veterinary Medical Association and is appropriate for the species, stages of development and size of the embryos. Eggs were obtained from Sunnyside Farms, Inc. (Beaver Dam, WI, United States) and incubated in the central auditory physiology laboratory at Northwestern University. For immunohistochemical experiments, chicken hatchlings of either sex were used. Eggs were obtained from Charles River Laboratories (Wilmington, MA, United States) and incubated in a Florida State University vivarium.

### Brainstem Slice Preparation

Ages for electrophysiological study were embryonic days (E) 19–21, when near-mature hearing ability of chickens is established. Briefly, the brainstem was dissected and isolated in oxygenated low-Ca^2+^ high-Mg^2+^ modified artificial cerebral spinal fluid (ACSF) containing the following (in mM): 130 NaCl, 2.5 KCl, 1.25 NaH_2_PO_4_, 26 NaHCO_3_, 3 MgCl_2_, 1 CaCl_2_, and 10 glucose. ACSF was continuously bubbled with a mixture of 95% O_2_/5% CO_2_ (pH 7.4, osmolarity 295-310 mOsm/l). The brainstem was blocked coronally, affixed to the stage of a vibratome slicing chamber (Ted Pella, Inc., Redding, CA, United States) and submerged in ACSF. Bilaterally symmetrical coronal slices were made (200 μm thick) and approximately seven slices containing NM were taken from caudal to rostral, roughly representing the low-to-high frequency regions, respectively. The caudal-most two to three slices were used in the current study (Wang et al., 2017).

Slices were collected in a custom holding chamber and allowed to equilibrate for 1 h at ∼22°C in aforementioned ACSF, but with 1 MgCl_2_ and 3 CaCl_2_ instead. Normal ACSF was continuously bubbled with a mixture of 95% O_2_/5% CO_2_ (pH 7.4, osmolarity 295–310 mOsm/l). Slices were transferred to a recording chamber mounted on an Olympus BX51W1 (Center Valley, PA, United States) microscope. The microscope was equipped with a CCD camera, 60× water-immersion objective and infrared differential interference contrast optics. The recording chamber was superfused continuously (Welco, Tokyo, Japan) at room temperatures (monitored continuously at ∼22°C, Warner Instruments, Hamden, CT, United States) in oxygenated normal ACSF at a rate of 1.5–2 ml/min. In a subset of experiments, recording temperature was increased to 35°C (see **Figure 5**).

### *In Vitro* Whole Cell Electrophysiology

Voltage- and current-clamp experiments were performed using an Axon Multiclamp 700B amplifier (Molecular Devices, Silicon Valley, CA, United States). Patch pipettes were pulled to a tip diameter of 1–2 μm using a P-97 flaming/brown micropipette puller (Sutter Instrument, Novato, CA, United States) and had resistances ranging from 3 to 6 MΩ. For voltage-clamp experiments of isolated K_V_ currents, the internal solution contained the following (in mM): 105 K-gluconate, 35 KCl, 1 MgCl_2_, 10 HEPES-K^+^, 5 EGTA, 4 4-Mg_2_ATP, 0.3 4-Tris_2_GTP, pH adjusted to 7.3–7.4 with KOH. The junction potential was ∼-10 mV and data were corrected accordingly. For voltage-clamp experiments of isolated Na_V_ currents, the internal solution was cesium-based and contained the following (in mM): 150 CsCl, 10 NaCl, 0.2 EGTA, 10 HEPES, pH adjusted to 7.3–7.4 with CsOH. The junction potential was ∼-3 mV and data were not corrected. The Cs-based internal solution was used to block K_V_ currents and reduce space-clamp issues. Series resistance was compensated for by ∼80% in all voltage-clamp recordings. For current-clamp experiments, the internal solution was the same as used for recording K_V_ currents. The junction potential (∼-10 mV) was not corrected for in our current-clamp experiments.

Pipettes were visually guided to the caudolateral region of NM, termed NMc, where neurons were identified and distinguished from surrounding tissue based on cell morphology, known structure, and location of the nucleus within the slice, as described in our recent study (Wang et al., 2017). After a GΩ seal was attained, membrane patches were ruptured and neurons were first held in the voltage-clamp mode of whole-cell configuration. A small hyperpolarizing (-1 mV, 30 ms) voltage command was presented at the beginning of each recorded trace to document and monitor whole-cell parameters (cell membrane capacitance, series resistance and input resistance). Neurons were included in the data analysis only if they had series resistances < 15 MΩ. For recording Na_V_ current, raw data was low-pass filtered at 5 kHz and digitized at 50 kHz using a Digidata 1440A (Molecular Devices). For recording K_V_ current and current-clamp experiments, raw data was low-pass filtered at 2 or 5 kHz and digitized at 20 or 50 kHz.

All experiments were conducted in the presence of picrotoxin (PTX, 100 μM, a GABA_A_ receptor antagonist), DL-2-amino-5-phosphonopentanoic acid (DL-APV, 100 μM, an NMDA receptor antagonist) and 6-Cyano-7-nitroquinoxaline-2, 3-dione (CNQX, 20 μM, an AMPA receptor antagonist). Isolated K_V_ currents were recorded in the presence of the Na_V_ channel blocker tetrodotoxin (TTx, 1 μM) and isolated Na_V_ currents were recorded with bath application of K_V_ channel blockers tetraethylammonium (TEA, 3 mM) and 4-AP (30 μM), along with CdCl_2_ (0.2 mM) to block calcium channels. Fluoxetine (Flx, 100 μM), a highly potent blocker of K_V_3.1-containing channels (Sung et al., 2008), was bath applied to estimate the ratio of K_V_3 mediated currents to the total K_V_ current. In a subset of experiments, TEA (1 mM) was substituted for Flx. Percent reduction of K_V_ current during application of Flx (*n* = 10) or TEA (*n* = 8) was compared at membrane voltages of -45, -10, and +20 mV. Since no difference was observed (*p* = 0.556, 0.449, and 0.593, respectively), data were pooled. Dendrotoxin (DTx, 0.1 μM), a potent blocker of K_V_1.1, K_V_1.2-containing channels, was bath applied to estimate the ratio of K_V_1 mediated currents. Guangxitoxin (GxTx, 100 nM), a highly specific blocker of K_V_2.1, K_V_2.2-containing channels (Liu and Bean, 2014), was used to estimate the ratio of K_V_2 mediated currents. Potassium leak currents were measured offline using the averaged responses to hyperpolarizing voltage commands from -80 to -90 mV as a baseline and were subtracted from the raw data.

Total K_V_ conductances (*G*_K_) and Na_V_ conductances (*G*_Na_) were obtained by the equation *I*_K/Na_ = *G*_K/Na_ (*V*_MEMBRANE_ – *E*_K/Na_). *I*_K_ and *I*_Na_ represent the potassium and sodium current measured in response to membrane voltage (*V*_MEMBRANE_), respectively. Based on our external and internal recording solutions, the reversal potential for K_V_ channels (*E*_K_) was -84 mV. The reversal potential for Na_V_ channels (*E*_Na_) was estimated by linear extrapolation from the current-voltage relationship of each individual neuron. In addition, current density (ρ_K/Na_) was calculated by normalizing isolated currents to the individual membrane capacitance. The voltage dependence curves of Na_V_ channel inactivation (*h*_Na_) were fitted using a Boltzmann function where h_Na_ = 1/[1+e^(VMEMBRANE - V_1/2_)/k^], in order to calculate half inactivation voltage (*V*_1/2_) and slope factor (*k*).

Na_V_ current data were obtained from the second and third most caudal slices, representing the majority of NMc1 neurons (Wang et al., 2017). Transient Na_V_ current (*I*_NaT_) obtained by step depolarization to -30 mV (holding voltage = -60 mV) was used for characterizing two Na_V_ current properties for individual NMc neurons: kinetics and reliability. Na_V_ current kinetics contains three variables: rise and fall rates, and half width. Rise and fall rates were defined as the maximal rate of rise and decay of *I*_NaT_, respectively. Half width was calculated as *I*_NaT_ duration measured at half of the maximum amplitude. In order to quantify *I*_NaT_ reliability, we applied the same voltage step over 30 repetitive trials (interpulse stimulus intervals = 2 s). Reliability was defined as the range of peak occurrences of 30 *I*_NaT_ evoked (Hong et al., 2016) and is a measure of peak latency jitter (i.e., larger jitter results in less reliable generation of Na_V_ current). The *I*_NaT_ kinetics were also obtained and averaged over these 30 trials.

To isolate and characterize the resurgent and persistent Na_V_ currents (*I*_NaR_ and *I*_NaP_, respectively) for NMc neurons regarding their relatively small amplitude, we ran the voltage-clamp protocols (see section “Results” for details) repetitively before and during application of TTx (1 μM). The currents shown in **Figure 7** were obtained by subtracting the TTx-insensitive current traces from the control traces. Capacitive currents generated during voltage-clamp recordings were blanked or reduced offline.

Under current-clamp mode, NMc neurons were held in whole-cell configuration at *I* = 0 for recording AP properties. APs were evoked by injecting a sustained current command of 200 pA (duration = 100 ms), and two variables regarding AP kinetics were measured before and during application of specific blockers (TEA or GxTx): AP half width and fall rate. Half width was quantified as AP duration measured at half of the maximum amplitude relative to the resting membrane potential. Fall rate was calculated as the maximal rate of decay in the AP repolarizing phase. Both AP properties were measured and averaged over 30 repetitive trials. In addition, we injected suprathreshold sinusoidal currents with the amplitude of 200 or 300 pA to characterize the frequency-firing responses of NMc neurons (Hong et al., 2016). The frequencies of sinusoidal currents were 5, 10, 40, 50, 75, 100, 150, and 200 Hz. Evoked APs per sinusoidal cycle (for simplicity, “APs per cycle”) were calculated as the number of APs divided by the total number of sinusoidal cycles and plotted as a function of stimulus frequency. Inter-spike interval (ISI) for responses at each frequency was calculated and plotted as histograms. We observed burst firing of NMc neurons to sinusoidal inputs of 5 and 10 Hz (i.e., generation of multiple APs per cycle, see **Figure 5**). Therefore, ISI was calculated for APs evoked within each sinusoidal cycle, while intervals between two consecutive cycles (mostly > 40 ms) were dismissed. By doing this, the histogram of ISI reflected the burst firing rate per cycle in response to sinusoidal stimulation of 5 and 10 Hz. In contrast, NMc neurons fired only one AP per sinusoidal cycle to input frequency > 40 Hz. The calculation of ISI was based on a previous study (Pilati et al., 2012), and thus ISI was defined as the interval between two consecutive APs.

### Data Analysis

**FIGURE 2:**
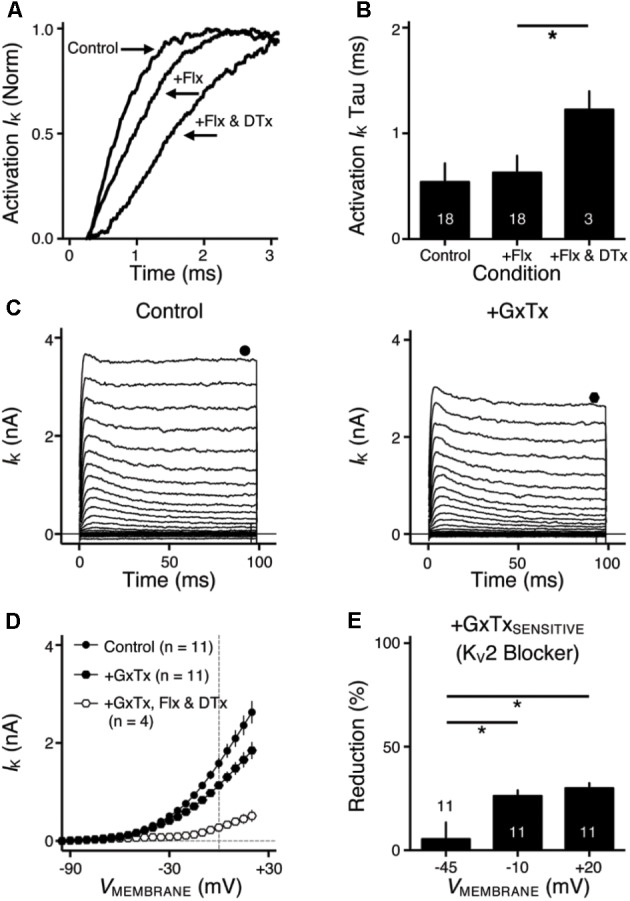
K_V_2 mediated current in NMc neurons. **(A)** Representative activation phase of K_V_ current traces (activation *I*_K_) with normalization (norm) in control and during drug application. Flx, fluoxetine (100 μM). DTx, dendrotoxin (0.1 μM). **(B)** Population data showing the time constant (tau) of K_V_ current activation phase. **(C)** Representative K_V_ current traces (*I*_K_) in control and during drug application. GxTx, Guangxitoxin (100 nM). Symbols (circle and hexagon) at the end of current traces represent time window of measured steady-state (SS) K_V_ currents. **(D)** Population data showing the relationship of steady-state (SS) K_V_ currents to membrane voltages (*V*_MEMBRANE_) in control and during drug application. **(E)** Population data showing the percent reduction in steady-state (SS) K_V_ currents due to the application of GxTx, at membrane voltages (*V*_MEMBRANE_) of +20, –10, and –45 mV. Error bar = standard error. ^∗^*p* < 0.05.

**FIGURE 3:**
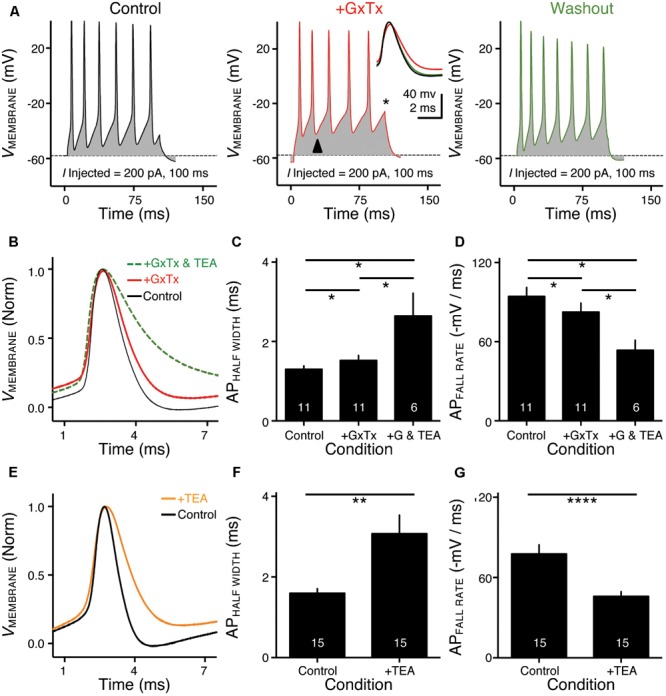
K_V_2- and K_V_3-containing channels regulate action potential (AP) kinetics for NMc neurons. **(A)** Representative membrane responses recorded from an NMc neuron in control, during application of GxTx and during washout process. The amplitude of current injection is 200 pA for 100 ms. Arrowhead points to the evident membrane depolarization during AP firing under GxTx. Asterisk denotes the failure of AP. Inset shows superimposed first APs under three conditions. **(B,E)** Representative APs with normalization (norm) in control and during drug application. GxTx, Guangxitoxin (100 nM). TEA, tetraethylammonium (1 mM). **(C,F)** Population data showing AP half width in control and during drug application. **(D,G)** Population data showing AP fall rate (in absolute value) in control and during drug application. G represents GxTx. Error bar = standard error. Numbers in bars represent sample size. ^∗^*p* < 0.05, ^∗∗^*p* < 0.01, ^∗∗∗∗^*p* < 0.0001.

**FIGURE 4:**
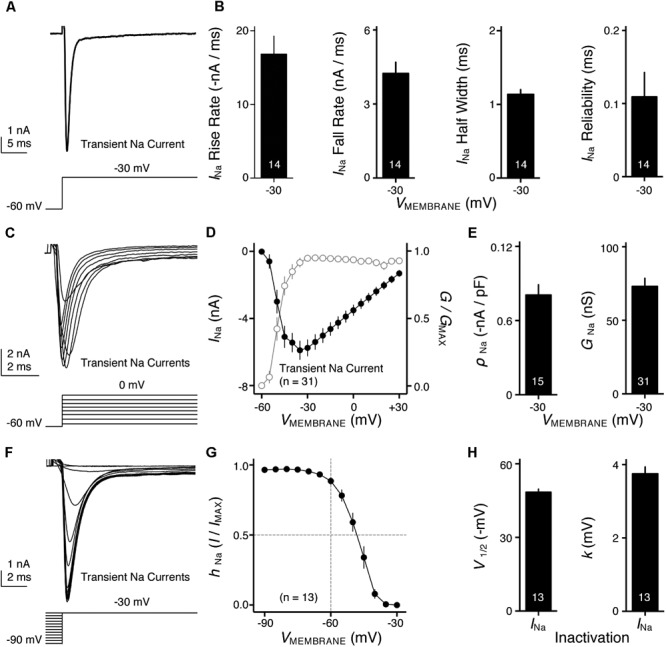
Voltage dependent sodium (Na_V_) current properties of NMc neurons. **(A)** Representative transient Na_V_ current traces (*I*_Na_) recorded from an NMc neuron, in response to step depolarization to –30 mV (holding voltage = –60 mV). **(B)** Population data showing the Na_V_ current (*I*_Na_) kinetics, including rise rate (in absolute value), fall rate and half width, along with reliability. These properties were measured at the membrane voltage (*V*_MEMBRANE_) of –30 mV. Numbers in bars represent sample size. **(C)** Representative transient Na_V_ current traces (*I*_Na_) in response to varying step depolarizations from –40 to 0 mV (Δ step = 5 mV, holding voltage = –60 mV). **(D)** Population data showing the Na_V_ current-voltage relationship (filled circles), along with normalized Na_V_ conductance (*G*/*G*_MAX_) as a function of membrane voltage (*V*_MEMBRANE_) for NMc neurons (open circles). **(E)** Population data showing the Na_V_ current density (ρ_Na_) and conductance (*G*_Na_) at the membrane voltage (*V*_MEMBRANE_) of –30 mV. **(F)** Representative transient Na_V_ current traces (*I*_Na_) in response to step depolarization to –30 mV following pre-pulse holding voltages clamped from –90 to –30 mV (Δ step = 5 mV). **(G)** Population data showing voltage dependence of Na_V_ current inactivation for NMc neurons. *h*_Na_ was calculated as the Na_V_ current recorded from each holding voltage (*I*) normalized to the maximum current across all trials (*I*_MAX_) and plotted as a function of the pre-pulse holding voltage. **(H)** Population data showing the half inactivation voltage (*V*_1/2_) and slope factor (*k*) for voltage dependence of inactivation. Error bar = standard error.

**FIGURE 5:**
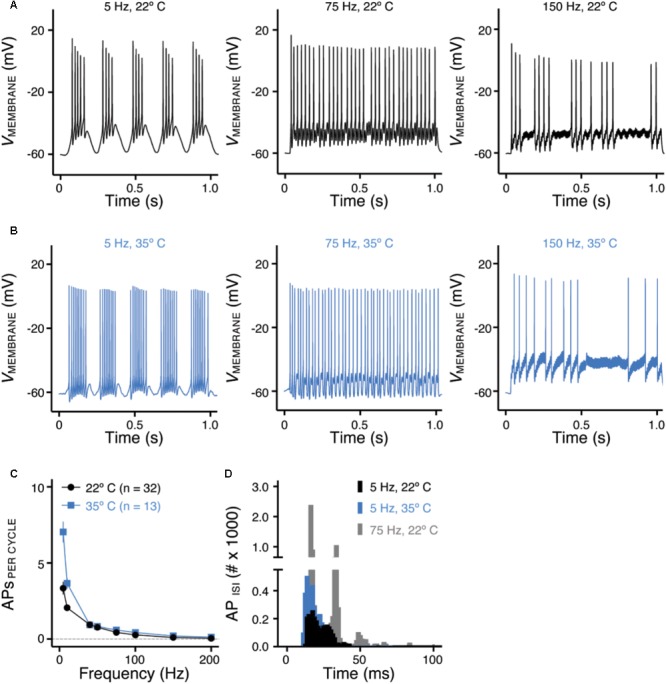
Frequency-firing pattern of NMc neurons to sinusoidal current injections. **(A,B)** Representative voltage traces recorded from NMc neurons in response to 5, 75, and 150 Hz sinusoidal current injections at 22°C **(A)** or 35°C **(B)** recording temperature. The strength of sinusoidal current injections is 200 or 300 pA. **(C)** Population data showing the evoked APs per sinusoidal cycle plotted as a function of stimulus frequency for NMc neurons. **(D)** Histogram of the inter-spike intervals (ISIs) calculated from APs in response to 5 and 75 Hz sinusoidal current injections. Error bar = standard error.

**FIGURE 6:**
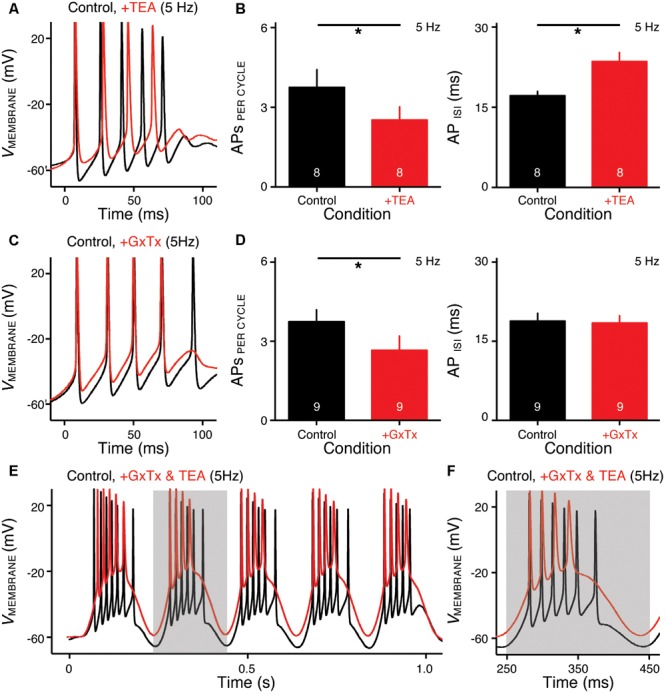
K_V_2- and K_V_3-containing channels regulate burst firing for NMc neurons. **(A,C)** Representative voltage responses to the first cycle of 5 Hz sinusoidal current injections in control (black) and during the application (red) of TEA **(A)** or GxTx **(C)**. TEA, tetraethylammonium (1 mM). GxTx, Guangxitoxin (100 nM). **(B,D)** Population data showing APs per cycle and inter-spike interval (ISI) in response to 5 Hz sinusoidal current injections, in control and during the application of TEA **(B)** or GxTx **(D)**. Error bar = standard error. Numbers in bars represent sample size. ^∗^*p* < 0.05. **(E,F)** Representative voltage responses recorded from an NMc neuron to 5 Hz sinusoidal current injections in control (black) and during dual drug application (red) of GxTx and TEA. Gray area of the second sinusoidal cycle in **(E)** was expanded and shown in **(F)**.

**FIGURE 7:**
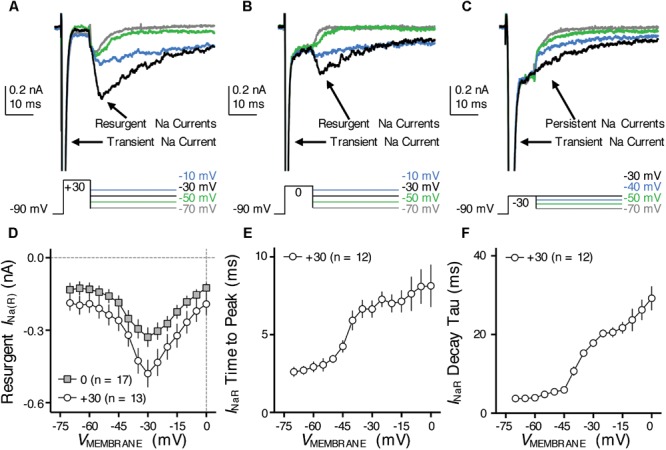
Resurgent Na_V_ current (*I*_NaR_) of NMc neurons. **(A–C)** Representative current traces in response to voltage-clamp protocols that elicit *I*_NaR_ (if any) shown below traces. The amplitude of the conditioning step is +30 mV in **(A)**, 0 mV in **(B)** and –30 mV in **(C)**. The duration of the conditioning step is 10 ms. **(D)** Population data showing the *I*_NaR_ amplitude plotted as a function of repolarizing membrane voltage (*V*_MEMBRANE_) for NMc neurons, in response to the conditioning step shown in **(A,B)**. **(E,F)** Population data showing the *I*_NaR_ time to peak **(E)** and decay time constant (tau, **F**) as a function of repolarizing membrane voltage (*V*_MEMBRANE_), in response to conditioning step of +30 mV. Error bar = standard error.

**FIGURE 8:**
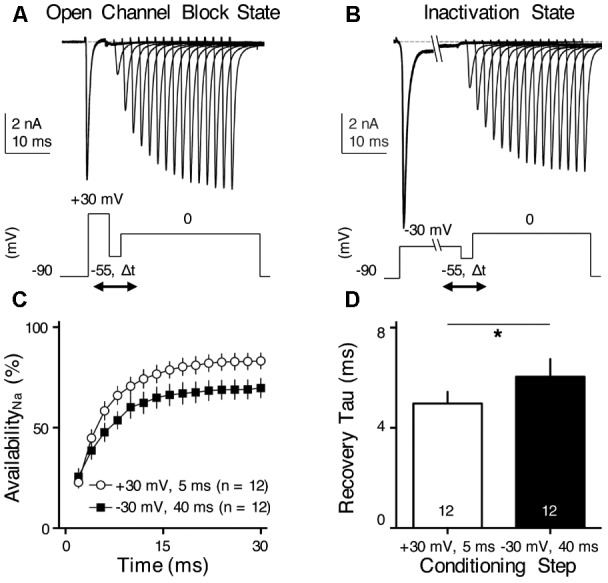
*I*_NaR_ helps increase Na_V_ channel availability and facilitates Na_V_ recovery. **(A,B)** Representative current traces in response to voltage-clamp protocols shown below traces. The conditioning step is +30 mV for 5 ms in **(A)** (Open Channel Block State) and –30 mV for 40 ms in **(B)** (Inactivation State). Δ*t* represents the varying recovery time, increasing from 2 to 30 ms in steps of 2 ms. **(C)** Population data showing the Na_V_ channel availability (%) as a function of recovery time. In order to calculate Na_V_ channel availability, a reference pulse to 0 mV was applied to NMc neurons (not shown in the figure). The amplitude of recovered Na_V_ current was first adjusted by subtracting the steady-state current that remained at the end of the conditioning step, before being normalized to the reference pulse. The recovery trajectory was fit by a single exponential, in order to obtain recovery time constant (tau) shown in **(D)**. **(D)** Population data showing the recovery time constant (tau) under two different condition states. Numbers in bars represent sample size. Error bar = standard error. ^∗^*p* < 0.05.

Recording protocols were written and run using Clampex acquisition and Clampfit analysis software (version 10.3; Molecular Devices, Silicon Valley, CA, United States). Statistical analyses and graphing protocols were performed using Prism (GraphPad versions 7.0b, La Jolla, CA, United States) and MATLAB (version R2014b; The MathWorks, Natick, MA, United States) software. Student *t*-tests or analysis of variance (ANOVA) with *post hoc* Bonferroni adjusted *t*-tests were used to determine significance, unless otherwise mentioned. The standard for significant differences was defined as *p* < 0.05. Numeric values in the texts and **Table 1** are reported as mean ± standard deviation (SD). Error bars in **Figures 1–8** represent standard error of the mean (SEM).

**Table 1:** Biophysical heterogeneity across the tonotopic axis in NM.

Properties	NMc (*n*)	M to HF NM^a^	*P, t*-test
K_V_ current properties			
Current at +20 mV (nA)	2.83 ± 0.90 (35)	6.24 ± 1.33 (39)	*P <*0.0001
Current density at +20 mV (nA/pF)	0.10 ± 0.65 (25)^∗^	0.26 ± 0.66 (15)^∗^	*P <*0.0001
Conductance at +20 mV (nS)	24.79 ± 7.90 (35)	54.74 ± 11.64 (39)	*P* < 0.0001
K_V_1 mediated current at +20 mV^b^	∼25%	∼49%	
K_V_2 mediated current at +20 mV^b^	∼30%	Minimal^c^	
K_V_3 mediated current at +20 mV^b^	∼40%	∼51%	
Transient Na_V_ current properties			
Max rise rate (nA/ms)	–16.83 ± 9.18 (14)	–10.55 ± 5.74 (11)	*P* = 0.059
Max fall rate (nA/ms)	4.24 ± 1.69 (14)	2.78 ± 1.12 (11)	*P* = 0.022
Half width (ms)	1.14 ± 0.24 (14)	1.13 ± 0.14 (11)	*P* = 0.934
Reliability range (ms)	0.11 ± 0.12 (13)	0.50 ± 0.42 (18)	*P* = 0.003
Current at -30 mV (nA)	–5.75 ± 2.68 (31)	–2.94 ± 0.88 (11)	*P* = 0.002
Current density at -30 mV (nA/pF)	–0.08 ± 0.03 (15)	–0.09 ± 0.05 (10)	*P* = 0.687
Conductance at -30 mV (nS)	73.11 ± 30.57 (31)	54.70 ± 13.59 (11)	*P* = 0.011
Inactivation *V*_1/2_ (mV)	–48.42 ± 4.30 (12)	–54.67 ± 3.77 (7)	*P* = 0.002
Slope factor *k* (mV)	3.75 ± 0.64 (12)	4.55 ± 0.70 (6)	*P* = 0.027
Resurgent Na_V_ current properties			
Max current (nA)	–0.49 ± 0.21 (13)	–0.81 ± 0.21 (17)	*P* = 0.0003
Time to peak (ms)^d^	6.65 ± 1.83 (12)	4.28 ± 0.70 (17)	*P* < 0.0001
Decay time constant (ms)^d^	17.82 ± 2.65 (12)	24.92 ± 6.71 (17)	*P* = 0.002
Persistent Na_V_ current properties			
Current at -30 mV (nA)^e^	–0.10 ± 0.03 (12)	–0.14 ± 0.05 (16)	*P* = 0.019

*^*a*^Data of mid- to high-frequency NM neurons are taken from Hong et al. (2016) for K_*V*_ and transient Na_*V*_ current properties, and Hong et al. (2017) for resurgent and persistent Na_*V*_ current properties. M, mid, HF = high-frequency.*

*^*b*^Percent relative to the total K_*V*_ current.*

*^*c*^From Kuba et al. (2015) and Hong et al. (2016).*

*^*d*^Time to peak and decay time constant were calculated at the repolarizing membrane voltage of -30 mV for both neuronal groups. The conditioning step is +30 mV for 10 ms.*

*^*e*^Persistent current amplitude was measured at the end of 100 ms repolarizing membrane voltage at -30 mV for both neuronal groups. The conditioning step is +30 mV for 10 ms.*

*^∗^Non-parametric Mann–Whitney U test was used to compare current density at +20 mV.*

### Reagents

All bath applied drugs were allowed to perfuse through the recording chamber for ∼5 min before subsequent recordings. DL-APV, CNQX and all other salts and chemicals were obtained from Sigma-Aldrich (St. Louis, MO, United States), PTX and Flx from Tocris (Ellisville, MO, United States), TTx, DTx, and GxTx from Alomone Labs (Jerusalem, Israel), and TEA from VWR (Radnor, PA, United States).

### Computational Modeling

**FIGURE 9:**
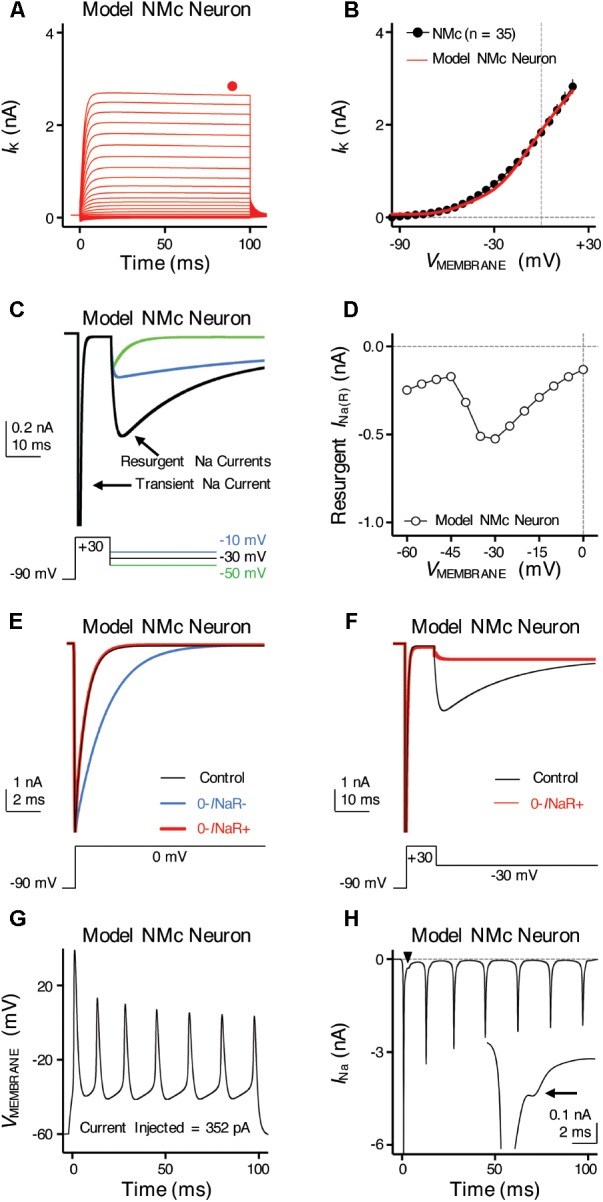
Simulations of ion channel properties and firing activity from model NMc neuron. **(A)** Simulated K_V_ current traces (*I*_K_) from model NMc neuron, in response to membrane voltages clamped from –100 to +20 mV (Δ step = 5 mV, duration = 100 ms). Steady-state (SS) K_V_ currents were measured at the end of current traces (filled circle). **(B)** Current-voltage relationship of simulated *I*_K_ from model NMc neuron (solid line), superimposed with experimental population data shown in **Figure 1E** (dots). **(C)** Simulated Na_V_ current traces in response to voltage-clamp protocols that elicit *I*_NaR_ shown below traces. **(D)** Current-voltage relationship of simulated *I*_NaR_ from model NMc neuron. **(E)** Simulated transient Na_V_ currents (*I*_NaT_) in response to step depolarization to 0 mV (holding voltage = –90 mV). *I*_NaT_ obtained under control condition is shown in black. Switching off *I*_NaR_ by setting the rate constant for the O–>OB transition, 𝜀, to zero resulted in considerable slowing of *I*_NaT_ decay (‘0-*I*NaR-’ condition, blue trace). *I*_NaT_ kinetics were restored by setting rate constant *O*_on_ to 2.15 ms^-1^ and *O*_off_ to 0.01433 ms^-1^ (‘0-*I*NaR+’ condition, red trace). **(F)** Simulated *I*_NaR_ in control (black) and in “0-*I*NaR+” condition (red). Removal of *I*_NaR_ in “0-*I*NaR+” condition has no effect on persistent Na_V_ current (*I*_NaP_). **(G)** Simulated AP firing from model NMc neuron in response to sustained current injection at 352 pA for 100 ms. **(H)** Simulated Na_V_ current trace underlying the firing activity shown in **(G)**. Arrowhead points to the area that was expanded in inset. Arrow points to the generation of *I*_NaR_ immediately after an *I*_NaT_.

Simulation of NMc electrical activity were performed in NEURON 7.1 (Hines and Carnevale, 1997) by employing a single-compartment model (**Table 2**). The NMc model was based on our NM model previously described and all ion currents were modeled using the same formalism (**Tables 2, 3**, Hong et al., 2017; Lu et al., 2017). Briefly, this model contains currents mediated by low- and high-voltage activated potassium (K^+^_LV A_ and K^+^_HV A_, respectively) channels, Na_V_, and passive leak channels. K^+^_LV A_, K^+^_HV A_, and passive leak channels were modeled in HH type formalisms (Rothman and Manis, 2003; Howard and Rubel, 2010; Hong et al., 2017; Lu et al., 2017). Na_V_ channel was modeled with a Markovian 13-state model, which generates the *I*_NaT_, *I*_NaP_, and *I*_NaR_ components simultaneously (Khaliq et al., 2003). All ion channel parameters were adjusted to match the experimental data obtained from E19–21 NMc neurons (**Table 3**). Parameters of K^+^_LV A_ and K^+^_HV A_ were adjusted such that the current-voltage curve of the model replicated that of the experimental data recorded from NMc neurons (see **Figures 9A,B**). Parameters of Na_V_ were adjusted such that spike threshold and amplitude were similar between the model and the experimental data obtained from NMc neurons. To switch off *I*_NaR_ without affecting the *I*_NaT_ and *I*_NaP_, we applied the method described by Magistretti et al. (2006) and Hong et al. (2017). Kinetic constants were modified as follows: the rate constant for the O->OB transition, 𝜀, was set to 0, *O*_on_, and *O*_off_ were increased to 2.15 and 0.01433 ms^-1^ to restore the kinetics of *I*_NaT_ and the amplitude of *I*_NaP_, respectively (see **Figures 9E,F**).

**Table 2:** Single compartment model.

Parameter	Value
Axial resistance	50 Ωcm
Temperature	22° C
*E*_Na_	47 mV
*E*_K_	–80 mV
Length	20 μm
Diameter	20 μm
*g*Leak	0.0004 S/cm^2^
*g*Na_V_	0.026 S/cm^2^
*g*K^+^_LV A_	0.00195 S/cm^2^
*g*K^+^_HV A_	0.0008 S/cm^2^

*Modified from Hong et al. (2017).*

**Table 3:** Model parameters and formulas.

Parameter	Value
***I*_NaV_**	**Markovian 13 states**
α	150exp(*V*/17) ms^-1^
β	3exp(-*V*/17) ms^-1^
γ	150 ms^-1^
δ	40 ms^-1^
𝜀	1.75 ms^-1^
ζ	0.035exp(-*V*/25) ms^-1^
*C*_on_	0.005 ms^-1^
*C*_off_	0.5 ms^-1^
*O*_on_	0.75 ms^-1^
*O*_off_	0.005 ms^-1^
a	(*O*_on_/*C*_on_)^1/4^
b	(*O*_off_/*C*_off_)^1/4^
T_0_	22°C
***I*_K+LV A_**	**Hodgkin–Huxley style**
*w*_∞_	1/(1+exp(-(*V*+67)/8)
*z*_∞_	1/(1+exp(-(*V*+71)/10)
*τ*_w_	(100/(6^∗^exp((*V*+60)/6)+16^∗^exp(-(*V*+60)/45))+1.5
τ_z_	(100/(exp((*V*+60)/20)+exp(-(*V*+60)/8))+50
T_0_	22°C
***I*_K+HV A_**	**Hodgkin–Huxley style**
*n*_∞_	1/(1+exp(-(*V*+35)/14)
*p*_∞_	1/(1+exp(-(*V*+71)/10)
τ_n_	(100/(11^∗^exp((*V*+60)/24)+21^∗^exp(-(*V*+60)/23))+0.7
τ_p_	(100/(4^∗^exp((*V*+60)/32)+5^∗^exp(-(*V*+60)/22))+5
T_0_	22°C

*V, C, and O denote voltage, closed and opened states, respectively. Modified from Hong et al. (2017).*

### Immunohistochemistry

For K_V_3.1b and K_V_2.2, chicken hatchlings (P2–14; *n* = 6) were transcardially perfused with 0.9% saline followed by 4% paraformaldehyde in 0.1 M phosphate buffer (PB). For Na_V_1.6, chicken hatchlings (P2–14; *n* = 4) were transcardially perfused with 0.9% saline followed by modified periodate–lysin–paraformaldehyde (PLP) fixative (ml/g body weight): 0.2% (w/v) paraformaldehyde, 2.7% (w/v) lysin HCl, 0.21% (w/v) NaIO_4_, and 0.1% (w/v) Na_2_HPO_4_ (Kuba et al., 2005). Following post-fix overnight in the same fixatives, brains were then transferred to 30% sucrose in PB for 3 days and sectioned in the coronal plane at 30 μm on a freezing sliding microtome. Each section was collected in 0.01 M phosphate buffered saline (PBS).

For K_V_3.1b immunostaining, free-floating sections were incubated with primary antibody (Sigma; AB5188) solutions diluted 1: 10,000 in PBS with 0.3% Triton X-100 overnight at 4°C. Sections were then incubated in a biotinylated IgG antibody (1:200; Vector Laboratories, Burlingame, CA, United States) diluted in PBS with 0.3% Triton X-100 for an hour at room temperature. After washing in PBS, sections were incubated in avidin–biotin–peroxidase complex solution (ABC Elite kit; Vector Laboratories) diluted 1:100 in PBS with 0.3% Triton X-100 for 1 h at room temperature. Sections were then washed in PBS and incubated for 3–5 min in 0.045% 3-3-diaminobenzidine (Sigma) with 0.03% hydrogen peroxide in PB. Sections were mounted on gelatin-coated slides and dehydrated, cleared, and cover slipped with Permount mounting medium (Fisher Scientific). The antibody has been fully characterized regarding its specificity of recognizing the chicken K_V_3.1b (Parameshwaran et al., 2001) and used for studying the chicken auditory brainstem (Lu et al., 2004).

Anti-Na_V_1.6 antibody was generously provided by Dr. Hiroshi Kuba at Kyoto University (Kuba et al., 2006). Following primary antibody incubation (1:1000 diluted in PBS with 0.3% Triton X-100), sections were incubated in Alexa-Fluor secondary antibodies (Life Technologies, Carlsbad, CA, United States) at 1:1000 overnight at 4°C. Sections were double stained with a somatodendritic marker, the microtubule-associated protein 2 (MAP2, Millipore; MAB3418) (Wang and Rubel, 2008). Sections were then mounted on gelatin-coated slides and cover slipped with Fluoromount-G mounting medium^®^ (Southern Biotech, Birmingham, AL, United States). The specificity of this antibody recognizing the chicken Na_V_1.6 was fully characterized (Kuba et al., 2006).

For K_V_2.2 immunostaining, a peptide containing the amino acids 47–60 (EVLWRTLDRLPtRTR) from the chicken K_V_2.2 (Accession number XM_003640825) was synthesized and used as antigen to immunize male rabbits (Thermo Fisher Scientific, Carlsbad, CA, United States). The generated antibody (K_V_2-19) was affinity purified and tested for specificity in western blots. 40 μg of protein lysate derived from chicken brainstem was used for western blot assay as previously described (Zorio et al., 2017). Free-floating sections were treated with 0.25% pepsin in 5 mM HCl for 15 min at 37°C, followed by primary antibody solution diluted 1: 5,000 in PBS with 0.3% Triton X-100 overnight at 4°C. Sections were incubated in Alexa-Fluor secondary antibodies, mounted, and cover slipped as described above.

### Quantification of Na_V_1.6 Immunoreactivity in the Axon Initial Segment

Three animals (P2, P4, and P11) were used for this analysis. Using an Olympus FV1200 confocal microscope, image stacks of different NM subregions were collected with 60× oil-immersion lens at a resolution of 0.1 μm per pixel at XY dimensions and with a Z interval of 0.5 μm. These imaging settings provide sufficient resolution for accurate identification and reconstruction of Na_V_1.6 immunoreactive segments. Olympus OIB image stacks were directly imported to Neurolucida (version 9.03; MBF Bioscience). Intact segments with both ends contained within the same stack were used for subsequent 3D reconstruction. The Na_V_1.6 immunoreactive segments were traced with lines through the center. Based on this reconstruction, the length is measured using Neurolucida Explorer (version 9.03; MBF Bioscience). No tissue shrinkage correction was applied. For each segment, the diameter was measured at three different locations of the middle portion of the segment using ImageJ software and averaged. As no differences were seen between ages from P2 to P11, the length and diameter of Na_V_1.6 immunoreactive segments from the same NM subregions were pooled across the three animals. These parameters were compared among subregions using one-way ANOVA with multiple comparisons using Prism software (**Table 4**). *P* < 0.05 was considered statistically significant. All data are shown as mean ± SD in the text and in **Figure 12**.

**Table 4:** Na_V_1.6^+^ fragment quantification.

Region	Fragment length/μm (*n*)	Fragment diameter/μm (*n*)
Rostral NM	13.32 ± 3.72 (23)	0.97 ± 0.24 (30)
NMcm^∗^	14.24 ± 3.97 (16)	1.00 ± 0.32 (23)
NMc1	22.41 ± 4.39 (51)	1.32 ± 0.35 (35)
NMc2	12.60 ± 4.15 (12)	0.86 ± 0.29 (21)
**ANOVA**		
*F*	39.98	38.77
*P*	<0.0001	<0.0001

*^∗^NMcm represents NM neurons located at caudomedial region adjacent to NMc, and do not show elaborate dendrites*.

## Results

Our previous study showed distinct active and passive membrane properties of NMc neurons (Wang et al., 2017). NMc neurons are more excitable (**Figure 1A**) than their higher-frequency counterparts (**Figure 1B**) and are capable of firing multiple APs to increasing current injection strength (**Figure 1C**). However, NMc neurons have slower AP kinetics and a significantly longer time constant; due in part to their higher input resistance. Here, we investigated K_V_ and Na_V_ channel properties underlying this unique physiology of NMc neurons. We compared these properties between NMc and mid- to high-frequency NM neurons (for simplicity, “adendritic NM neurons”) (Hong et al., 2016). In addition, we characterized the frequency-firing pattern of NMc neurons to sinusoidal current injections, as well as the role of K_V_ and Na_V_ channels in shaping this firing pattern phenotype. Electrophysiological data were obtained from 210 NMc neurons at E19-21.

### Distinct K_V_ Current Properties of NMc Neurons

We previously showed that low- (K^+^_LV A_) and high-voltage (K^+^_HV A_) activated potassium channels regulate neuronal excitability and AP kinetics in NM, respectively (Hong et al., 2016). Therefore, we hypothesized that NMc neurons have significantly less K^+^_LV A_ and K^+^_HV A_ currents (especially K_V_3 and K_V_1 mediated currents) than adendritic NM neurons. To test our hypothesis, voltage-clamp recordings of K_V_ current were performed. NMc neurons were held at -70 mV before being clamped by 100 ms steps to membrane voltages ranging from -100 to +20 mV, with a 5 mV increment. Steady-state K_V_ current was measured at the end of each current trace and plotted as a function of membrane voltage (**Figure 1D**, filled circle and **Figure 1E**). We observed significantly lower amount of total K_V_ currents for NMc neurons, which was 2.83 ± 0.90 nA at +20 mV, as compared to 6.24 ± 1.33 nA for adendritic NM neurons (*p* < 0.0001, **Table 1**). **Figure 1F** shows the population data of K_V_ current density and total conductance at +20 mV for NMc neurons and when compared to adendritic neurons, both of them are significantly smaller (*p* < 0.0001, **Table 1**). Taken together, NMc neurons showed lower amount of K_V_ current, as a result of reduced total conductance and lower density of K_V_ channels.

We further characterized the ratio of K_V_ current that is mediated by different K_V_ channel subunits. Flx or 1 mM TEA was bath applied to block K_V_3-containing channels (**Figure 1G** *left* and *middle*, **Figure 1H**). Data using two different drugs were pooled. The percent of current reduction during drug application was calculated at three membrane voltages: -45, -10, and +20 mV. In line with K_V_3 function as K^+^_HV A_ channels, the greatest reduction (∼40%) was observed at positive membrane voltage (i.e., +20 mV), and the percent change significantly decreased from +20 to -45 mV (**Figure 1I**, *p* = 0.0002, repeated measures ANOVA). We observed a minor amount of K_V_ current reduced at -45 mV (∼9%), likely due to the non-specific blockade of other K_V_ subunits by Flx or TEA (Tytgat et al., 1997; Johnston et al., 2010). Subsequent application of DTx was used to block K_V_1-containing channels (**Figure 1G** *right*, **Figure 1H**). As expected, the greatest current reduction during DTx application was found at -45 mV (**Figure 1J**), because K_V_1-containing channels are activated around the resting membrane potential (Johnston et al., 2010; Hong et al., 2016). At +20 mV, DTx sensitive current accounted for only ∼25% of total K_V_ currents, although this difference between voltages is not significant (**Figure 1J**, *p* = 0.078).

We next investigated which K_V_ channel subunits likely mediate the remaining 35% (**Figure 1E**) of total K_V_ currents at +20 mV. We noticed that dual application of Flx and DTx slowed down the activation kinetics of K_V_ current at +20 mV (**Figure 2A**). Current traces +20 mV in **Figure 2A** were taken from **Figure 1G** and were normalized to highlight the difference in their activation phase. A single exponential was fit to the dominant component of activation phase and a time constant (tau) of K_V_ activation was calculated (Rathouz and Trussell, 1998; Liu and Bean, 2014). We found a significant increase in activation time constant after dual application of Flx and DTx (*p* = 0.018, *post hoc* Bonferroni adjusted *t*-tests), but not with Flx only (*p* = 0.717, **Figure 2B**). Application of DTx has no effect on activation kinetics of K_V_ current (Owen et al., 1997) and changes in activation time constant is likely attributable to properties of remaining currents. K_V_3- and K_V_1-containing channels are well known to activate in an extremely fast manner (Rathouz and Trussell, 1998; Johnston et al., 2010), and thus potential K_V_ channel subunits mediating the remaining current are likely presented with significantly slower kinetics. In addition, half activation voltage (*V*_1/2_) of the remaining current is ∼-11 mV. These observations of the remaining current largely resembled the K_V_2 mediated current reported elsewhere, such as neurons in medial nucleus of trapezoid body (MNTB) and in superior cervical ganglion (Johnston et al., 2008; Liu and Bean, 2014). Therefore, we applied a specific K_V_2 blocker (Guangxitoxin, GxTx) on NMc neurons and it reduced ∼30% of total K_V_ currents at +20 mV (**Figures 2C–E**). Due to the high-voltage activation property of K_V_2-containing channels, maximal reduction occurred at +20 mV, whereas little amount of current was blocked at -45 mV (**Figure 2E**, *p* = 0.007, repeated measures ANOVA). Finally, triple application of GxTx, Flx, and DTx abolished the majority of steady-state K_V_ current in NMc neurons (**Figure 2D**). In summary, our results revealed a unique combination of K_V_ channel subunits in NMc neurons; K_V_1-, K_V_2-, and K_V_3-containing channels mediated ∼25, ∼30, and ∼40% of total current at positive membrane voltage, respectively. This combination of K_V_ channel subunits is in distinct contrast to that of adendritic NM neurons, in which K_V_1- and K_V_3-containing channels each account for approximately half of total current, while the presence of K_V_2 mediated current is minimal (Kuba et al., 2015; Hong et al., 2016). The comparison also confirmed that K_V_1 and K_V_3 mediated currents in NMc neurons are significantly less than adendritic NM neurons (**Table 1**).

### Role of K_V_3- and K_V_2-Containing Channels in Regulating AP Kinetics of NMc Neurons

K_V_3- and K_V_2-containing channels are both K^+^_HV A_ channels and contribute to the repolarizing phase of APs (Rudy and McBain, 2001; Johnston et al., 2010). Blockade of these channels leads to a significant increase in AP duration and a slowing of AP falling phase (Liu and Bean, 2014; Kimm et al., 2015; Hong et al., 2016). However, maximal activation of K_V_2-containing channels only occurs at the late phase of the AP repolarization due to their slow kinetics, and thus these channels are suggested to play a secondary role in regulating AP kinetics compared to other fast-activating K_V_ channels in mammalian neurons (Johnston et al., 2010; Liu and Bean, 2014; Kimm et al., 2015). Based on previous studies, we hypothesized that K_V_3- and K_V_2-containing channels both regulate AP repolarizing kinetics for NMc neurons, but to a different extent. To test this hypothesis, we injected a sustained (100 ms) current command with the strength of 200 pA to evoke APs in NMc neurons. This current strength is well above the average threshold current of NMc neurons, as shown in our recent study (∼40 pA) (Wang et al., 2017). Bath application of GxTx resulted in a depolarized after hyperpolarization time period following AP firing (**Figure 3A** *left* and *middle*, arrowhead) and reduced the number of APs (**Figure 3A** *middle*, asterisk). Washout of GxTx restored the AP response properties to control values (**Figure 3A** *left* and *right*). Analysis of first AP revealed a slight but significant increase in AP half width (**Figure 3A** *middle*, inset, **Figures 3B,C**, *p* = 0.006, paired *t*-test). A more dramatic increase in half width was observed after subsequent application of 1 mM TEA (**Figures 3B,C**, *p* = 0.028). Similarly, AP fall rate reduced significantly during GxTx application (*p* = 0.001), but the reduction after dual application of GxTx and TEA was most prominent (*p* = 0.003, **Figures 3B,D**). When we applied TEA alone, the percent changes in AP half width and fall rate were significant (*p* = 0.002 and *p* < 0.0001, respectively) and larger than those with GxTx application (**Figures 3E–G**). For example, GxTx application increased AP half width by ∼16% on average while TEA application by ∼87% (*p* = 0.001). Taken together, K_V_3- and K_V_2-containing channels both regulate AP repolarizing kinetics for NMc neurons, but K_V_3-containing channels play a more dominant role, probably as a result of the following facts: first, K_V_3-containing channels activate much earlier than K_V_2-containing channels during short AP period; second, K_V_3 mediated current is larger than K_V_2 mediated current in NMc neurons (see above).

### Distinct Na_V_ Current Properties of NMc Neurons

Na_V_ channels play a critical role in AP generation and thus may be another factor that subserves AP firing pattern of NMc neurons (Eijkelkamp et al., 2012). To test this prediction, we first profiled the properties of Na_V_ current for NMc neurons. Na_V_ current data were obtained from the second and third most caudal slices, representing the majority of NMc1 neurons (Wang et al., 2017). **Figure 4A** shows a transient Na_V_ current (*I*_NaT_) in response to step depolarization to -30 mV (holding voltage = -60 mV). This current was used to characterize the kinetics (i.e., rise rate, fall rate, and half width) and reliability of *I*_NaT_ for individual NMc neuron (see section “Materials and Methods”), the population data of which are shown in **Figure 4B**.

We next characterized the voltage dependence of *I*_NaT_ activation and inactivation for NMc neurons. Individual NMc neuron was held at -60 mV before being clamped at membrane voltages from -60 to +30 mV, with a 5 mV increment. **Figure 4C** shows the representative current traces in response to varying membrane voltages. The amplitudes of *I*_NaT_ and normalized conductance were plotted as a function of membrane voltage in **Figure 4D** (filled and open circles, respectively). As indicated by **Figure 4D**, Na_V_ current density and total conductance were near maximum at -30 mV, the population data of which are shown in **Figure 4E**. Furthermore, to characterize the inactivation property of *I*_NaT_, NMc neurons were held at membrane voltages ranging from -90 to -30 mV with a 5 mV increment, followed by a step depolarization to -30 mV (**Figure 4F**). The amplitude of *I*_NaT_ generated from each holding voltage was normalized to the maximal amplitude across all trials and plotted as a function of holding voltage (**Figure 4G**). A Boltzmann function was fit for each normalized curve and half inactivation voltage (*V*_1/2_) and slope factor (*k*) were calculated (**Figure 4H**, see section “Materials and Methods”). Comparisons of the aforementioned properties between NMc neurons and their higher-frequency counterparts revealed several significant differences (**Table 1**). First, NMc neurons showed faster *I*_NaT_ fall rate (*p* = 0.022) and generated *I*_NaT_ more reliably (i.e., smaller reliability range, *p* = 0.003). It should be noted that reliability (and jitter) were compared near membrane voltage that elicited the largest *I*_NaT_. This membrane voltage maximized the electrochemical driving force for sodium ions and thus ensured fair comparisons between NMc and adendritic NM neurons. Second, NMc neurons (mainly NMc1) displayed larger *I*_NaT_ (*p* = 0.002). This result is consistent with our immunohistochemical findings (see below and section “Discussion”). Third, this amplitude difference was due to a significantly higher total conductance of NMc neurons (*p* = 0.011) but not to Na_V_ current density (*p* = 0.687). Forth, NMc neurons showed a more depolarized voltage dependence of inactivation, demonstrated by a significantly less negative *V*_1/2_ (*p* = 0.002). Finally, the slope factor of the inactivation curve (*k*) was smaller in NMc neurons, indicating a steeper inactivation curve than that reported from adendritic NM neurons (*p* = 0.027) (Hong et al., 2016). These differences might reflect distinction in Na_V_ α-subunits and/or the auxiliary β-subunits (see section “Discussion”).

### Frequency-Firing Pattern of NMc Neurons to Sinusoidal Current Injections

Numerous studies have demonstrated the important role of K_V_ and Na_V_ channels in regulating neuronal AP firing patterns (For review see Bean, 2007). To further investigate the function of these channels across the tonotopic axis, we used sinusoidal current injections to document frequency-firing pattern of NMc neurons. Based on their K_V_ and Na_V_ current properties, we hypothesized distinct frequency-firing patterns to sinusoidal inputs of NMc neurons compared to higher-frequency NM neurons. We applied suprathreshold sinusoidal current injections (200/300 pA) with frequencies ranging from 5 to 200 Hz and calculated APs per cycle (see section “Materials and Methods”). NMc neurons generated burst firing of APs in response to the rising phase of 5 Hz sinusoidal cycles (**Figure 5A**, *left* and **Figure 5C**). Burst firing was also observed at 10 Hz with reduced APs per cycle (**Figure 5C**). In response to 40 Hz sinusoidal current injections, NMc neurons fired an AP per cycle on average, but APs per cycle dropped to ∼0.5 at 75 Hz (**Figure 5A**, *middle* and **Figure 5C**), which indicates that NMc neurons were only able to fire at ∼37.5 Hz on average to the 75 Hz input (i.e., AP failures on every other cycle). As the stimulus frequency increased, APs per cycle reduced dramatically and only a few APs were observed in response to 150 Hz stimulation (**Figure 5A**, *right* and **Figure 5C**). These results demonstrate that NMc neurons act as a low-pass filter in response to sinusoidal current injections and fire optimally to stimulation < 40 Hz. It should be noted that burst firing was also observed to near-threshold sinusoidal current injections. Supplementary Figure S1 shows two representative NMc neurons with threshold current of 20 pA (Supplementary Figures S1A_1_,B_1_). Both NMc neurons fired bursts of APs to a 5 Hz current injection just above threshold (25 and 50 pA, Supplementary Figures S1A_2_,B_2_, respectively).

We further calculated the inter-spike intervals (ISIs) for APs generated in response to sinusoidal current injections. For AP responses to low-frequency stimulations (i.e., 5 and 10 Hz), only the spike intervals within the burst firing of each cycle were calculated (see section “Materials and Methods”). **Figure 5D** shows the histogram of ISIs for 5 and 75 Hz. The ISI histogram for 75 Hz is multimodal. The first and the largest peak of the histogram corresponds to ISI ∼13 ms, which represents the average interval between two consecutive APs. The second peak corresponds to ISI ∼27 ms, which is approximately twice as large as the ISI for the first peak and represents the average interval of two APs with a failure in between. Following this order, the third and the forth peak (though small) represent the average interval with two and three failures in between, respectively. The ISI histogram for 5 Hz is relatively bimodal, with the first and the largest peak superimposing the first peak of 75 Hz. This indicates that NMc neurons burst fired at ∼75 Hz. The second peak corresponds to ISI ∼22 ms, representing ∼45 Hz. Therefore, in response to 5 Hz sinusoidal current injections, NMc neurons generated burst firing at each cycle in relatively fast frequencies between 45 and 75 Hz.

In a subset of experiments, we recorded the frequency-firing pattern of NMc at near physiological temperature (i.e., 35°C). Burst firing to stimulations of 5 and 10 Hz became more robust at the higher temperature, as demonstrated by two major changes. First, we observed a significant increase in the number of APs per cycle (**Figure 5B**, *left* and **Figure 5C**). Second, the histogram of ISI for burst firing to 5 Hz stimulation peaked at 11–14 ms, corresponding to a more rapid firing rate of 71–91 Hz compared to room temperature (22°C, **Figure 5D**). In contrast, responses to stimulus frequencies greater than 40 Hz did not show significant differences in the number of APs per cycle (**Figure 5B**, *middle, right* and **Figure 5C**) or firing rate (data not shown). In summary, the number of APs per cycle and ISI at different recording temperatures indicate that NMc neurons are responsive to slow rising depolarization.

This functional phenotype of NMc neurons to sinusoidal inputs is in stark contrast to adendritic NM neurons, which show band-pass filter characteristic of firing pattern (Hong et al., 2016). Adendritic NM neurons do not fire APs to 5 and 10 Hz stimulation. However, this can be reversed and made similar to the firing pattern of NMc neurons with blockade of K^+^_LV A_ channels in a model NM neuron (Lu et al., 2017). The filtering function of adendritic NM neurons is due to the fast activation of K^+^_LV A_ channels that shunt the membrane depolarization induced by slow rising stimulation, such as sinusoidal current injection of 5 or 10 Hz (Lu et al., 2017). Additionally, adendritic NM neurons are able to follow 75 Hz stimulation continuously for 1 s in a one-to-one fashion, the firing capability of which can be reduced by blocking K^+^_HV A_ channels (Hong et al., 2016). Therefore, we conclude that the functional phenotype of NMc neurons is a combinatory result of their reduced K^+^_LV A_ (for burst firing to low-frequency stimulations) and K^+^_HV A_ conductances (for filtering out sinusoidal input with frequency > 40 Hz).

### Role of K_V_3- and K_V_2-Containing Channels in Regulating Low-Frequency Burst Firing for NMc Neurons

NMc neurons burst fired at 45–75 Hz to sinusoidal current injections of 5 Hz, a feature that is not present in adendritic NM neurons (Hong et al., 2016) and may be relevant to processing extremely low frequency information (i.e., infrasound). Therefore, we next characterized the ion channel properties that shape burst firing for NMc neurons. Based on the aforementioned role of K_V_3- and K_V_2-containing channels on AP kinetics, we hypothesized that these channels contribute to shaping burst firing in response to 5 Hz sinusoidal current injections. To test this hypothesis, we used TEA (1 mM) and GxTx to block K_V_3- and K_V_2-containing channels, respectively. **Figures 6A,C** show the representative voltage responses to the first cycle of 5 Hz stimulation before and during drug application. APs per cycle and ISIs were calculated under each condition. When bath applying TEA to block K_V_3-containing channels, we observed a significant reduction in APs per cycle (**Figures 6A,B** *left, p* = 0.012, paired *t*-test). In addition, ISIs increased significantly, indicating a significant slowing in the burst-firing rate (**Figures 6A,B** *right, p* = 0.003). Similarly, when bath applying GxTx to block K_V_2-containing channels, we also observed a significant reduction in APs per cycle (**Figures 6C,D** *left, p* = 0.042). However, ISIs did not change (**Figures 6C,D** *right, p* = 0.169). This result is likely attributable to two counteractive effects of K_V_2-containing channels on repetitive firing: first, activation of K_V_2-containing channels results in profound after hyperpolarization, which facilitates the recovery of Na_V_ channels and thus promotes repetitive firing; second, the slow kinetics of K_V_2 deactivation prolong the refractory period and thus impede repetitive firing (Johnston et al., 2010; Liu and Bean, 2014). Blockade of K_V_2-containing channels depolarized the membrane but also likely shortened the refractory period after APs. In summary, K_V_3-containing channels significantly regulate the bursting APs per cycle and firing rate, while K_V_2-containing channels only show a significant effect on the bursting APs per cycle. It should be noted that AP reduction in both cases was due to the failure of generating the last AP per cycle, indicating increased membrane depolarization when K_V_ channels are blocked.

In a subset of experiments, we were able to bath apply GxTx and TEA sequentially (*n* = 6 neurons). As we expected, NMc neurons with blockade of both K_V_3- and K_V_2-containing channels showed largely depolarized voltage responses as compared to those in control (**Figures 6E,F**). However, dual drug application did not abolish burst firing for the majority of NMc neurons (five out of six neurons). To our surprise, NMc neurons were able to fire multiple APs even when membrane voltages were depolarized enough to inactivate most Na_V_ channels (see **Figures 4G, 6F**). This result suggests that there are other factor(s) that promote the generation of burst firing in NMc neurons, even without profound after hyperpolarization.

### Resurgent Na_V_ Current of NMc Neurons

One such factor, as we hypothesized, is the resurgent Na_V_ current (*I*_NaR_) induced by a specific “open channel block state” that is indigenous to the Na_V_ channel (Grieco et al., 2005). This hypothesis is based on numerous previous studies that showed *I*_NaR_ being the key factor responsible for high-frequency firing and burst generation (Raman and Bean, 2001; Enomoto et al., 2007; Kim et al., 2010). Our recent study reported the presence and important function of *I*_NaR_ in mid- to high-frequency NM neurons (Hong et al., 2017). Therefore, to test our hypothesis, we first examined whether NMc neurons presented with *I*_NaR_ using classic voltage-clamp protocols (Raman and Bean, 1997). NMc neurons were held at -90 mV before application of a depolarizing step to +30 mV for 10 ms. This is referred to as the conditioning step. Next, NMc neurons were repolarized to membrane voltages ranging from -70 to 0 mV (Δ step = 5 mV) in order to elicit *I*_NaR_ (if any). With this protocol, we observed robust generation of *I*_NaR_ in NMc neurons (**Figure 7A**). The amplitude of *I*_NaR_ was measured and plotted as a function of repolarizing membrane voltage (**Figure 7D**). The voltage dependence of *I*_NaR_ in NMc neurons displayed a typical “V” shape that peaked at -30 mV, similar to those reported in mammalian neurons (Lewis and Raman, 2014). In addition, the maximal amplitude of *I*_NaR_ in NMc neurons was usually less than 1 nA and much smaller than the amplitude of *I*_NaT_ (see **Figures 4D, 7D**). This property also closely resembled that observed in other auditory brainstem neurons (Leao R.N. et al., 2006; Kim et al., 2010), including adendritic NM neurons (Hong et al., 2017).

Previous studies show that the amplitude of *I*_NaR_ is dependent on the level of conditioning step (Raman and Bean, 2001). The more positive the conditioning step is (e.g., +30 mV), the more likely to elicit larger *I*_NaR_. This is because the mechanism of the open channel block state (which is responsible for generating *I*_NaR_) competes against the classic inactivation mechanism that is induced by the cytoplasmic linker between III and IV domains of Na_V_ channels. More positive voltage steps help condition Na_V_ channels toward an open channel block state (and thus larger *I*_NaR_). We next examined whether the level-dependence property was also presented in NMc neurons by switching the step depolarization from +30 mV to 0 and -30 mV (duration = 10 ms). We still observed *I*_NaR_ in response to the conditioning step of 0 mV for 10 ms, but the *I*_NaR_ amplitude was generally smaller than using the conditioning step of +30 mV (**Figure 7B**). However, the *I*_NaR_ current-voltage relationship remained similar despite of different conditioning levels (i.e., both peaked at -30 mV, **Figure 7D**). Finally, no detectable *I*_NaR_ was observed in NMc neurons when using a more negative conditioning step (i.e., -30 mV, **Figure 7C**). Instead, large steady-state persistent Na_V_ currents (*I*_NaP_) were evident following the repolarization. These results further confirmed the similarity of *I*_NaR_ properties between NMc and other neurons reported elsewhere.

With respect to the kinetics of *I*_NaR_, two variables were calculated (1) time to peak, defined as the amount of time taken from the onset of repolarization to the peak of *I*_NaR_ and (2) decay time constant (tau), calculated by fitting a single exponential to the decay phase of *I*_NaR_. The conditioning step of +30 mV was used for calculation of *I*_NaR_ kinetics. Both variables were plotted as a function of repolarizing membrane voltage (**Figures 7E,F**). Similar to mammalian neurons (Lewis and Raman, 2014), both time to peak and decay time constant for NMc neurons increased gradually as the repolarizing membrane voltage became less negative. In summary, our results suggest that *I*_NaR_ is a common feature shared in the avian auditory brainstem, and a highly conserved property across different biological structures and species (Lewis and Raman, 2011).

Nevertheless, when comparing the *I*_NaR_ properties between NMc and adendritic NM neurons, we noticed several differences (**Table 1**) (Hong et al., 2017). First, the maximal *I*_NaR_ in NMc neurons was significantly smaller and accounted for only ∼60% of the maximal *I*_NaR_ in adendritic NM neurons. Second, the *I*_NaR_ voltage dependence in NMc neurons shifted toward the positive direction by ∼10 mV and peaked at -30 mV, whereas *I*_NaR_ peaked at -40 mV for adendritic NM neurons (Hong et al., 2017). Third, time to peak, which indicates the rate of open channel blocker unbinding from Na_V_ α-subunits, was larger in NMc neurons. This difference further suggests a higher affinity of open channel blocker to α-subunits in NMc neurons than in adendritic NM neurons. Finally, decay time constant (tau) was smaller in NMc neurons. Decay time constant depends on both the unbinding rate of open channel blocker and after the displacement of open channel blocker; the rate of α-subunits entering the classic inactivated or closed state (depending on membrane voltage). For NMc neurons, despite their longer time to displace the open channel blocker, shorter decay time constant suggests that their α-subunits entered the inactivated or closed state significantly faster than those in adendritic NM neurons. This result is consistent with the significantly larger fall rate of *I*_NaT_ in NMc neurons (see **Figure 4**). In addition, we also measured the amplitude of *I*_NaP_ at the end of 100 ms repolarization for NMc neurons and it was significantly smaller than adendritic neurons. Taken together, our comparisons revealed that *I*_NaR_ properties, including the amplitude, voltage dependence and kinetics, are surprisingly differentiated across tonotopic regions – a result that has not been reported in the auditory system.

### Role of *I*_NaR_ in Regulating Burst Firing for NMc Neurons: Experimental Results

We hypothesized that *I*_NaR_ with its underlying open channel blocker plays an important role in regulating burst firing in response to low-frequency stimulation for NMc neurons. Based upon this hypothesis, we predicted that the activation of open channel blocker would promote the Na_V_ channel availability and facilitate recovery from depolarization, which is ultimately important for burst firing at a relatively fast rate of 45–75 Hz. To test this prediction, we applied two voltage-clamp protocols that were previously used in mammalian and adendritic NM neurons (Raman and Bean, 2001; Patel et al., 2015; Hong et al., 2017). In the first protocol, NMc neurons were held at -90 mV before application of a conditioning depolarization to +30 mV for 5 ms (**Figure 8A**). According to the aforementioned observations (see **Figure 7**), this conditioning step maximized the occupancy of open channel blocker (referred here as “Open Channel Block State”). Next, the membrane voltage was set at -55 mV in order for NMc neurons to recover (average resting membrane potential = -50 mV without the correction of junction potential) (Wang et al., 2017). The recovery time varied from 2 to 30 ms with a 2 ms increment. Finally, a step depolarization to 0 mV was applied to evoke an *I*_NaT_. The second protocol was similar except for the conditioning step, which was -30 mV for 40 ms in order to maximize the occupancy of the classic inactivation gate (referred here as “Inactivation State,” **Figure 8B**). **Figures 8A,B** show representative *I*_NaT_ with varying amplitudes after the recovery under the two different conditions. In order to determine Na_V_ channel availability, we calculated the normalized ratio, which indicates the amount of available Na_V_ channels after the varying recovery period. To do this, we ran a reference pulse to 0 mV (holding voltage = -90 mV) prior to the implementation of the two protocols described above. The recovered *I*_NaT_ amplitude was normalized to this “reference amplitude” and plotted as a function of the recovery time (**Figure 8C**). As shown by two different recovery trajectories, the availability of Na_V_ channels in Open Channel Block State became significantly larger than in Inactivation State when recovery time was increased beyond 4 ms. The recovery trajectory was fit with a single exponential in order to obtain a recovery time constant (tau). We found that the Open Channel Block State significantly shortened the recovery time constant and facilitated the recovery of Na_V_ channels (**Figure 8D**, *p* = 0.018, paired *t*-test). These results suggest that *I*_NaR_ may help burst firing for NMc neurons.

### Role of *I*_NaR_ in Regulating Burst Firing for NMc Neurons: Computational Results

To further examine the role of *I*_NaR_ in regulating burst firing, we built a computational model for NMc neurons. This model is based on our previous studies (Hong et al., 2017; Lu et al., 2017), with parameters adjusted to match the experimental data obtained from NMc neurons (**Tables 2, 3**). **Figure 9A** shows K_V_ current traces of the model NMc neuron in response to membrane voltages from -100 to +20 mV, in steps of 5 mV. The steady-state K_V_ current was measured at the end of each trace and plotted as a function of membrane voltage. As shown in **Figure 9B**, the K_V_ current-voltage relationship of model NMc neuron matched the experimental data. When we applied voltage-clamp protocol with the conditioning step of +30 mV for 10 ms, model NMc neuron generated *I*_NaR_ during repolarizations with similar amplitude and voltage dependence of the experimental data (**Figures 9C,D**). The generation of *I*_NaR_ was based on Markovian 13-state Na_V_-channel model, which sets O->OB (i.e., open channel block) transition as a major exit path from the open state (Khaliq et al., 2003; Magistretti et al., 2006). In order to remove *I*_NaR_, we set the rate constant 𝜀 of this transition to zero. This modification, however, significantly slowed down the falling phase of *I*_NaT_ (**Figure 9E**, ‘0-*I*NaR-’ condition, blue trace), because removing one of the major exit paths resulted in the slower speed of channels exiting the open state. Therefore, we next increased the rate constant *O*_on_ to 2.15 ms^-1^ and *O*_off_ to 0.01433 ms^-1^ to restore the normal decay kinetics of *I*_NaT_ and amplitude of *I*_NaP_, respectively (**Figures 9E,F**, ‘0-*I*NaR+’ condition, red trace) (Hong et al., 2017). After these two modifications, *I*_NaR_ was successfully eliminated (**Figure 9F**). “0-*I*NaR+” condition was used to characterize the spiking activity of model NMc neuron without *I*_NaR_.

When switching to current-clamp mode, spiking activity of the model NMc neuron (in control) closely resembled the experimental results. The model neuron showed similar repetitive firing pattern to experimental data, in response to sustained current injection (**Figures 1A, 9G**). **Figure 9H** shows the Na_V_ current underlying this repetitive firing for the model NMc neuron. Interestingly, our modeling data closely resembled previous reports in cerebellar Purkinje cells, when repetitive APs were used as voltage commands and the underlying Na_V_ currents were recorded in voltage-clamp mode (also referred as ‘AP clamp’) (Raman and Bean, 1997, 1999). This is especially true when we observed an obvious inward-going component immediately after the first AP (**Figure 9H**, arrowhead and inset, arrow). This small inward current, based on previous AP-clamp studies, is the *I*_NaR_ (Raman and Bean, 1997; Raman et al., 2000; Do and Bean, 2003; Enomoto et al., 2006).

**FIGURE 10:**
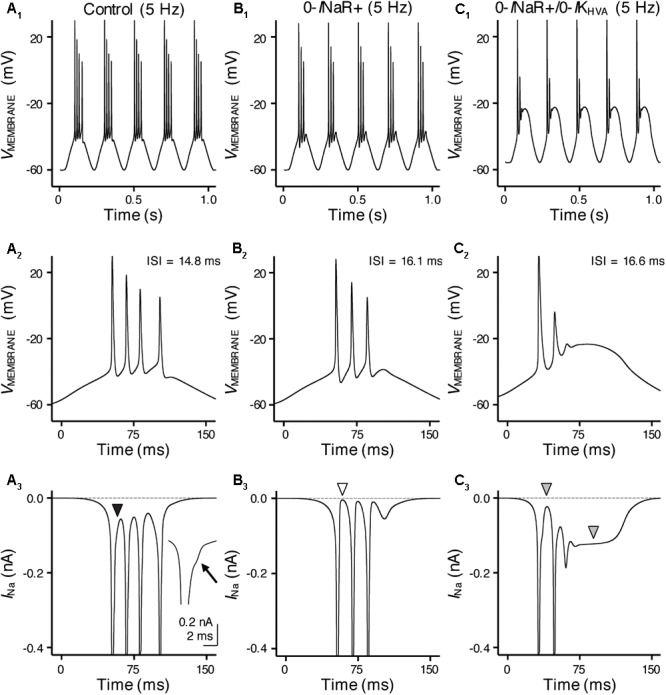
*I*_NaR_ and high-voltage activated potassium (K^+^_HV A_) channels synergistically promote burst firing for model NMc neuron. **(A_1_–C_1_)** Simulated voltage responses from model NMc neuron to 5 Hz sinusoidal current injections under three different conditions: control **(A_1_)**, with removal of *I*_NaR_ (‘0-*I*NaR+’, **B_1_**) and with removal of both *I*_NaR_ and K^+^_HV A_ conductances (‘0-*I*NaR+/0-*I*K_HV A_’, **C_1_**). The strength of sinusoidal current injections is 352 pA. **(A_2_–C_2_)** The expansion of simulated voltage responses to the first cycle of sinusoidal current injections, under three different conditions. The ISI represents the time difference between the first and second APs. **(A_3_–C_3_)** The expansion of simulated Na_V_ currents underlying the burst firing shown in **(A_2_–C_2_)**, respectively. Black arrowhead in **(A_3_)** points to the generation of *I*_NaR_ between APs. Arrow points to the generation of *I*_NaR_. White arrowhead in **(B_3_)** points to zero *I*_NaR_. Gray arrowheads in **(C_3_)** point to the generation of *I*_NaP_.

Our model NMc neuron also generated similar frequency responses to sinusoidal current injections. For example, **Figure 10A_1_** shows burst firing of the model neuron to a 5 Hz sinusoidal current injection. The model neuron generated 4 APs per cycle, which is around the average of our experimental data (see **Figure 5C**). **Figures 10A_2_,A_3_** show the expansion of voltage responses of the first sinusoidal cycle and the underlying Na_V_ current, respectively. The ISI of burst firing for model neuron was ∼14.8 ms, which is also similar to our experimental results (see **Figure 5D**). Additionally, we observed small inward-going Na_V_ currents between spikes (**Figure 10A_3_**, black arrowhead and inset, arrow), which were probably *I*_NaR_ (see below).

With removal of the *I*_NaR_ (0-*I*NaR+), burst firing of model NMc neuron was reduced to 3 APs per sinusoidal cycle with prolonged ISI of ∼16.1 ms (**Figures 10B_1_,B_2_**). It is worth noting that the small inward-going Na_V_ currents between spikes observed in control were no longer visible in the “0-*I*NaR+” condition (**Figure 10B_3_**, white arrowhead), confirming that these currents were indeed the *I*_NaR_. The reduction in APs was due to Na_V_ channel inactivation that resulted in the failure of generating a final AP per cycle. These results revealed that *I*_NaR_ plays a role in promoting the burst-like firing of APs per stimulus cycle and the overall firing rate of NMc neurons. In addition, we further set K^+^_HV A_ conductances to zero in “0-*I*NaR+” condition (termed 0-*I*NaR+/0-*I*K_HV A_). With removal of both K^+^_HV A_ and *I*_NaR_, the model NMc neuron was only able to generate 2 APs per cycle and ISI was further extended to ∼16.6 ms (**Figures 10C_1_,C_2_**). The membrane voltage was largely depolarized due to the lack of K_V_ conductances. In this case, no *I*_NaR_ was able to activate between spikes at the depolarized membrane voltages (**Figure 10C_3_**, first gray arrowhead) and thus the model NMc neuron entered depolarization block after firing two APs. This observation is different from the result shown in **Figure 6E**, where NMc neurons were able to burst fire at largely depolarized membrane voltages, likely due to *I*_NaR_. It is also worth noting that a relatively large amount of *I*_NaP_ was activated and sustained during depolarization block (**Figure 10C_3_**, second gray arrowhead), which suggests that *I*_NaP_ contributed minimally to subsequent AP firing for NMc neurons. Taken together, our computational results demonstrated that *I*_NaR_ and K^+^_HV A_ channels work synergistically to promote burst firing for NMc neurons in response to low-frequency stimulations.

### Distribution of K_V_ and Na_V_ Channels in NMc

**FIGURE 11:**
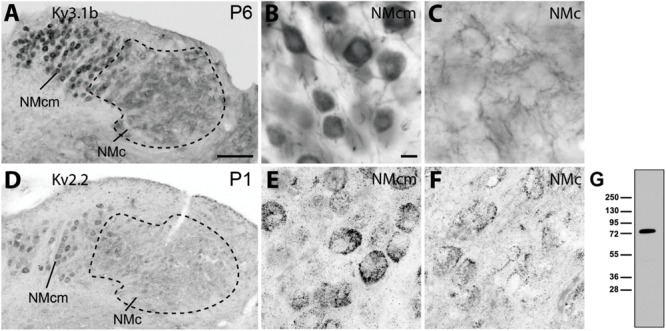
Immunoreactivity of K_V_3.1b and K_V_2.2 in NMc. **(A–C)** Low- and high-magnification images of K_V_3.1b immunoreactivity in the caudal NM. The dashed line in **(A)** outlines the border of NMc. **(D–F)** Low- and high-magnification images of K_V_2.2 immunoreactivity in the caudal NM. The dashed line in **(D)** outlines the border of NMc. **(G)** K_V_2.2 antibody validation by western blot. NM, nucleus magnocellularis; NMcm, caudomedial NM; NMc, caudolateral NM. Scale bars = 100 μm in **(A)** (applies to **A,D**); 10 μm in **(B)** (applies to **B,C,E,F**).

To confirm the expression of K_V_ channels in NMc, we performed immunocytochemistry using antibodies recognizing different K_V_ channels. Consistent with a previous study (Parameshwaran et al., 2001) K_V_3.1b immunoreactivity was identified throughout NM. At the caudal level (**Figure 11A**), NMc displayed a distinct distribution pattern as comapred to the neighboring NMcm where neurons do not have dendrites (Wang et al., 2017). NMcm was characterized with strong somatic immunostaining while NMc contained primarily neuropil staining (**Figures 11B,C**). The custom-made anti-K_V_2.2 antibody recognized a single band in western blot at the molecular weight of approximate 80 kDa (**Figure 11G**). This antibody revealed somatic staining in both NMc and NMcm with varied levels of neuropil staining (**Figures 11D–F**).

**FIGURE 12:**
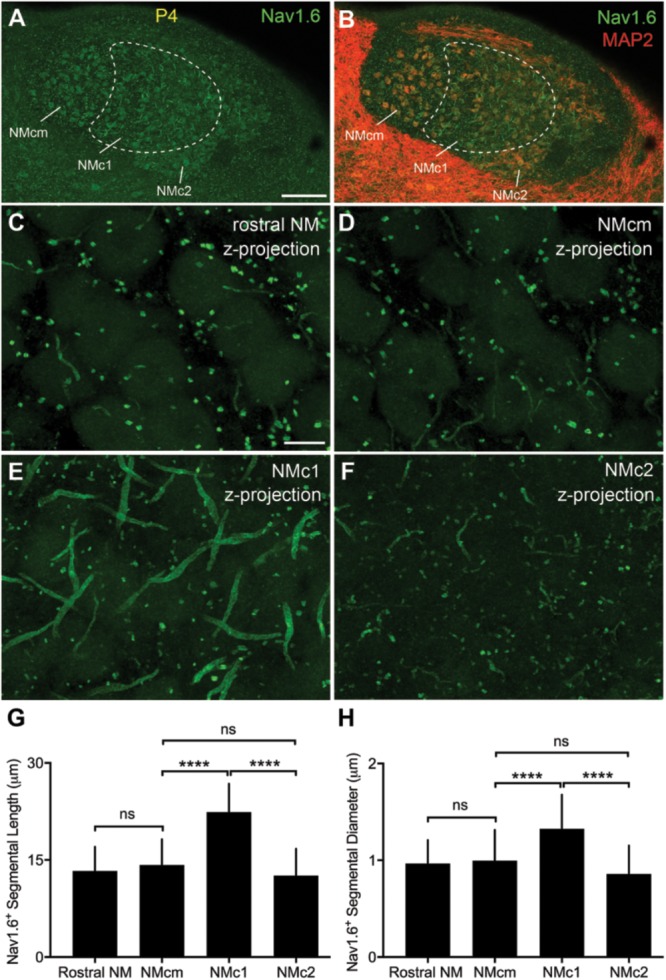
Na_V_1.6 immunoreactivity in NM. **(A,B)** Low-magnification images of Na_V_1.6 immunoreactivity in the caudal NM containing NMcm, NMc1 and NMc2. Sections were counterstained with MAP2 (red channel in **B**). To visualize MAP2 staining in NM, the image is saturated in surrounding areas with higher intensities of immunoreactivity than NM. Dashed lines outline the border of NMc1. **(C–F)** High power images with z-projection showing the immunoreactivity of Na_V_1.6 in the rostral NM **(C)**, NMcm **(D)**, NMc1 **(E)**, and NMc2 **(F)**, respectively. **(G,H)** Bar charts demonstrating the length **(G)** and diameter **(H)** of Nav1.6 stained segments in various NM subregions. NM, nucleus magnocellularis; NMcm, caudomedial NM; NMc1, caudolateral NM subregion 1; NMc2, caudolateral NM subregion 2. Scale bars = 100 μm in **(A)** (applies to **A,B**); 10 μm in **(C)** (applies to **C–F**). ^∗∗∗∗^*P* < 0.0001; ns, not significant.

Strong Na_V_1.6 immunoreactivity was observed throughout NM as bright punctate and short segments (**Figures 12A,B**). As described in our previous study (Hong et al., 2017), Na_V_1.6 segments were localized in NM axons that can be traced back to the cell bodies. Interestingly, we identified notably larger size of Na_V_1.6 immunoreactive segments in NMc1, one subregion of NMc (Wang et al., 2017), as compared to the remaining NM (**Figures 12C–F**). One-way ANOVA analysis confirmed significantly different lengths and diameters of Na_V_1.6 segments across different NM subregions (**Table 4**). Multiple comparisons further revealed that the length and diameter of Na_V_1.6 immunoreactive segments were significantly larger in NMc1 than NMc2 and NMcm as well as the rostral NM (**Figures 12G,H** and **Table 4**). These observations likely underlie the aforementioned larger *I*_NaT_ recorded from NMc1 region. We also suggest that the low-frequency NM region previously reported by Kuba and Ohmori (2009) – showing longer Na_V_1.6 labeled axonal segments – likely represents the NMc1 region.

## Discussion

Our results revealed diverse ion channel properties for neurons responsible for encoding low frequency sounds. NMc neurons, which process the lowest sound frequencies for chickens, showed significantly lower amount of K_V_1 and K_V_3 currents but more K_V_2 current than their higher-frequency counterparts. Despite their larger amplitude of *I*_NaT_, NMc neurons presented with significantly smaller *I*_NaR_. In line with these findings, immunochemistry showed K_V_2 immunoreactivity in NMc region and longer Na_V_1.6-containing segments along the axons of NMc1 neurons. Additionally, NMc neurons were most responsive to low-frequency sinusoidal current injections, burst firing of which was regulated differentially by K_V_3-, K_V_2-containing channels along with *I*_NaR_.

### K_V_ Channel Gradient Along Tonotopic Axis Is Conserved Across Species

In auditory brainstem neurons of both mammals and avians, APs generated by neurons in the low-frequency region are presented with wider duration, elevated excitability and lower timing reliability (Fukui and Ohmori, 2004). K_V_3-containing channels play an important role in shaping AP duration and thus promote firing at high-rates, whereas K_V_1-containing channels lower excitability, shorten the membrane time constant and reduce AP jitter (Wang et al., 1998; Johnston et al., 2010; Hong et al., 2016). Based on their specialized functions, one would expect that auditory neurons processing low frequencies show reduced level of K_V_3 and K_V_1 expression. Indeed, in mammalian MNTB, K_V_3.1 immunoreactivity is reduced in the low-frequency region (Strumbos et al., 2010). This is also true in NM (Parameshwaran et al., 2001). In our preparation, we observed reduced somatic levels of K_V_3.1b immunoreactivity in NMc (see **Figure 11**). Additionally, the level of K_V_1.1 mRNA is lowest toward the caudal pole of NM (Fukui and Ohmori, 2004). In line with immunochemistry findings, low-frequency MNTB neurons show less K_V_3 and K_V_1 currents (Brew and Forsythe, 2005). Here, we report similar results in NM. At positive membrane voltages, the amplitude of K_V_3 current is ∼1.13 nA for NMc neurons (i.e., 40% of 2.83 nA), while ∼3.18 nA for mid- to high-frequency NM neurons (**Table 1**). The amplitude of K_V_1 current is <1 nA compared to ∼3.06 nA in higher-frequency NM regions (Hong et al., 2016).

In contrast to K_V_3 and K_V_1, the tonotopic differentiation of other K_V_ subunits is less explored. A previous study in rat MNTB found a gradient of slow-kinetic K_V_ current that is reversed to the known gradients of K_V_3 and K_V_1, i.e., larger current toward the low-frequency region (Brew and Forsythe, 2005). A follow-up study confirmed that this K_V_ current is mediated by K_V_2 subunits (Johnston et al., 2008). The K_V_2 gradient found in mammalian auditory brainstem is also present in NM. In mid- to high-frequency NM neurons, K_V_ current was nearly reduced to zero when bath applying both DTx and TEA (Kuba et al., 2015; Hong et al., 2016). This differed in NMc neurons where a relatively large amount of K_V_ currents remained (see **Figure 1**); the majority of which was sensitive to the K_V_2 channel blocker, GxTx (see **Figure 2**). In summary, previous and current studies indicate that tonotopic differentiations of K_V_1, K_V_2, and K_V_3 in auditory brainstem are conserved properties across different species.

### *I*_NaR_ Properties Are Differentiated Along Tonotopic Axis of NM

*I*_NaR_ has been reported in numerous mammalian neurons, including auditory structures like the calyx of Held and MNTB principle neurons (Lewis and Raman, 2014). Our recent study showed that this current is also present in mid- to high-frequency NM neurons and thus *I*_NaR_ is another property conserved among mammals and birds and across different structures (Hong et al., 2017). However, it was not clear whether *I*_NaR_ properties show a tonotopic gradient. Our results in the current study demonstrated that *I*_NaR_ properties are differentiated along the tonotopic axis of NM. NMc neurons showed significantly smaller *I*_NaR_ with more depolarized voltage dependence (i.e., by ∼10 mV) compared to their higher-frequency counterpart. These differences may underlie distinct *I*_NaR_ function across tonotopic regions. For example, mid- to high-frequency NM neurons are able to repolarize the membrane quickly during high-frequency firing (i.e., up to 200 Hz), due to their large K_V_ conductances (Hong et al., 2017). Therefore, more hyperpolarized *I*_NaR_ voltage dependence in these neurons may be preferential for the maximal activation of *I*_NaR_. The removal of *I*_NaR_ in the model NM neuron reduced its high-frequency firing (Hong et al., 2017). In contrast, during burst firing of NMc neurons to low-frequency stimulations, the membrane voltage depolarized gradually (see **Figures 5, 6**). Thus, more depolarized *I*_NaR_ voltage dependence may optimize the activation of *I*_NaR_. Removal of *I*_NaR_ in the model NMc neuron led to reduction in burst-like firing of APs per sinusoidal cycle and a slower firing rate (see **Figure 10**). Taken together, tonotopic differentiation of *I*_NaR_ properties likely contributes to different firing activity for neurons processing different sound frequencies.

Distinct *I*_NaR_ properties along tonotopic axis are likely due to different Na_V_ α-subunits and/or β-subunits. However, both NMc and higher-frequency NM neurons showed extensive Na_V_1.6 expression, which raises the possibility that the auxiliary β-subunits might show different expression along tonotopic axis. This speculation is partially supported by previous findings from heterologous expression system, where the expression of β1-, β2-, and β4-subunits shows different effects on Na_V_ current properties, including *I*_NaT_, *I*_NaR_, and *I*_NaP_ (Qu et al., 2001; Aman et al., 2009). Particularly, β4-subunits have been proposed as the open channel blocker that induced *I*_NaR_ in cerebellar Purkinje cells, granule cells and dorsal root ganglion neurons (Grieco et al., 2005; Bant and Raman, 2010; Barbosa et al., 2015). However, the expression of β-subunits has not been characterized in the auditory system, except for a recent finding of β4-subunits in the spiral ganglion neurons of the auditory nerve and calyx of Held at MNTB (Berret et al., 2016; Browne et al., 2017). Future experiments will test this speculation.

### Functional Implication of Burst Firing and Underlying Mechanisms for NMc Neurons

The NM neurons receive inputs from auditory nerve and encode temporal information of sound by firing APs that “lock” to a specific phase, referred to as “phase locking.” Previous modeling studies demonstrated that NM neurons with different characteristic frequencies develop distinct strategies to improve or preserve phase locking abilities (Kuba and Ohmori, 2009; Oline et al., 2016; Lu et al., 2017). High-frequency neurons should only receive a small number of inputs (e.g., <3) in order to maintain relatively good phase locking, whereas low-frequency neurons can receive more inputs in order to improve phase locking (Oline et al., 2016). Anatomical evidences support this idea. Mid- to high-frequency NM neurons receive a few endbulb of Held synapses from the auditory nerve, while NMc neurons form numerous bouton synapses on their extensive dendrites (Jhaveri and Morest, 1982a,b; Wang et al., 2017). Regarding the physiology, converging inputs from multiple bouton synapses can reduce AP jitter and thus improve phase locking for NMc neurons. However, the resultant excitatory postsynaptic potential (EPSP) has a significantly slower rise phase due to the summation process (Kuba and Ohmori, 2009). In addition to input convergence, low-frequency NM neurons have larger NMDA receptor (NMDA-R) current compared to mid- and high-frequency NM due to a greater expression of GluN2B-containing receptors (Lu and Trussell, 2007). NMDA-Rs that contain the GluN2B subunit generate excitatory postsynaptic currents (EPSCs) with slow kinetics (Sanz-Clemente et al., 2013; Sanchez et al., 2015). Finally, according to behavior experiments, chickens can hear sound frequencies as low as 2 Hz (Hill et al., 2014). These studies indicate that low-frequency NMc neurons are exposed to slow rising stimulus with long wavelengths. However, we acknowledge that our experimental procedures (i.e., sinusoidal stimulation) take into account compromise when attempting to mimic *in vivo* conditions and caution must be taken when interpreting the data in a biological context. Nonetheless, we propose that using sinusoidal current injection can serve as a valid tool to investigate a neuron’s response to inputs with varying rise rates and wavelengths, and to better understand the underlying mechanisms. This is especially important for studying biophysical properties across tonotopic regions.

The aforementioned slower EPSP and EPSC are problematic for auditory neurons for several reasons. First, slow depolarization can activate a large amount of K_V_ currents, especially K^+^_LV A_, which quickly repolarize and shunt the membrane before reaching the threshold for Na_V_ channel activation. Second, slow depolarization can induce closed-state inactivation of Na_V_ channels that further raises the activation threshold. Both situations prevent neurons from firing APs and thus may cause a loss of sound information. To overcome this, NMc neurons develop specialized ion channel properties from mid- to high-frequency NM neurons. First, NMc neurons are presented with significantly lower K_V_ conductances, especially K^+^_LV A_ current. Although the exact contribution of distinct dendritic morphology and distribution patterns of K_V_ channels to K_V_ conductances require further determination. Second, NMc neurons (mainly NMc1) show larger Na_V_ current with higher channel density in the axons. Significantly larger Na_V_1.6 immunoreactive segments of NMc1 neurons may serve as one biochemical substrate underlying this larger Na_V_ current. The inactivation curve of Na_V_ channels also shifts toward depolarized direction to minimize closed-state inactivation. These results support the idea that NMc neurons, because of their numerous inputs and dendritic architecture, favor slow rates of depolarization and it is their distinct synaptic and intrinsic ion channel properties that allow them to establish their functional phenotypes that differ from neurons across the tonotopic axis.

Our recent modeling study demonstrated that burst firing to low-frequency sinusoidal current injections would occur as a consequence when K^+^_LV A_ current was reduced (Lu et al., 2017). Burst firing may be more preferential to encode low-frequency information, though the relationship of our results to that of an *in vivo* situation remains unclear. However, it is interesting to note that low-pass filter properties of NMc neurons reported here demonstrate some similarity to previous *in vivo* recordings (Warchol and Dallos, 1989). In their study, Warchol and Dallos (1989) showed that 60% of low-frequency NM neurons displayed tuning curves resembling low-pass filter functions, i.e., lowest threshold observed for sound frequencies from 10 to 50 Hz. How do NMc neurons stay responsive to such low frequencies? Generation of burst firing, as demonstrated in the current study may be one possibility. In addition, due to the long wavelength of low-frequency sound, several studies argued that auditory brainstem neurons might employ distinct strategies to encode interaural time difference (ITD) and to localize low-frequency sound, especially regarding animals with small heads (McAlpine et al., 2001; Carr and Koppl, 2004). For example, the preferential azimuth for individual sound localization neurons in both mammals and birds is identified by the peak slope of their ITD-function response curve (i.e., the most drastic change in the response), instead of their peak response to differences in interaural timing cues. Although it is difficult to relate this idea to the current findings, burst firing may promote the steepness of the response and help optimize low-frequency sound localization abilities. Future studies should be directed at addressing these possibilities. Our results suggest that burst firing and its underlying ion channel mechanisms may reflect a strategy adopted by NMc neurons in order to accurately encode low-frequency sound and may be an evolutionally conserved mechanism that subserves similar auditory-related functions across species.

## Author Contributions

All authors approved the final version of the manuscript and agree to be accountable for all aspects of the work. All persons designated as authors qualify for authorship, and all those who qualify for authorship are listed. HH, TL, XW, DZ, YW, and JS designed the study. HH performed the electrophysiology experiments at Northwestern University. TL performed the computational modeling experiments at Northwestern University. XW, DZ, and YW performed the immunocytochemical experiments at Florida State University. HH, TL, XW, DZ, YW, and JS analyzed/interpreted data and wrote the manuscript.

## Conflict of Interest Statement

The authors declare that the research was conducted in the absence of any commercial or financial relationships that could be construed as a potential conflict of interest.
