# Supplementary figures and images for: Diverse Intrinsic Properties Shape Functional Phenotype of Low-Frequency Neurons in the Auditory Brainstem
Source: Front Cell Neurosci. 2018 Jun 26;12:175. doi: 10.3389/fncel.2018.00175 (PMC6028565; doi:10.3389/fncel.2018.00175)

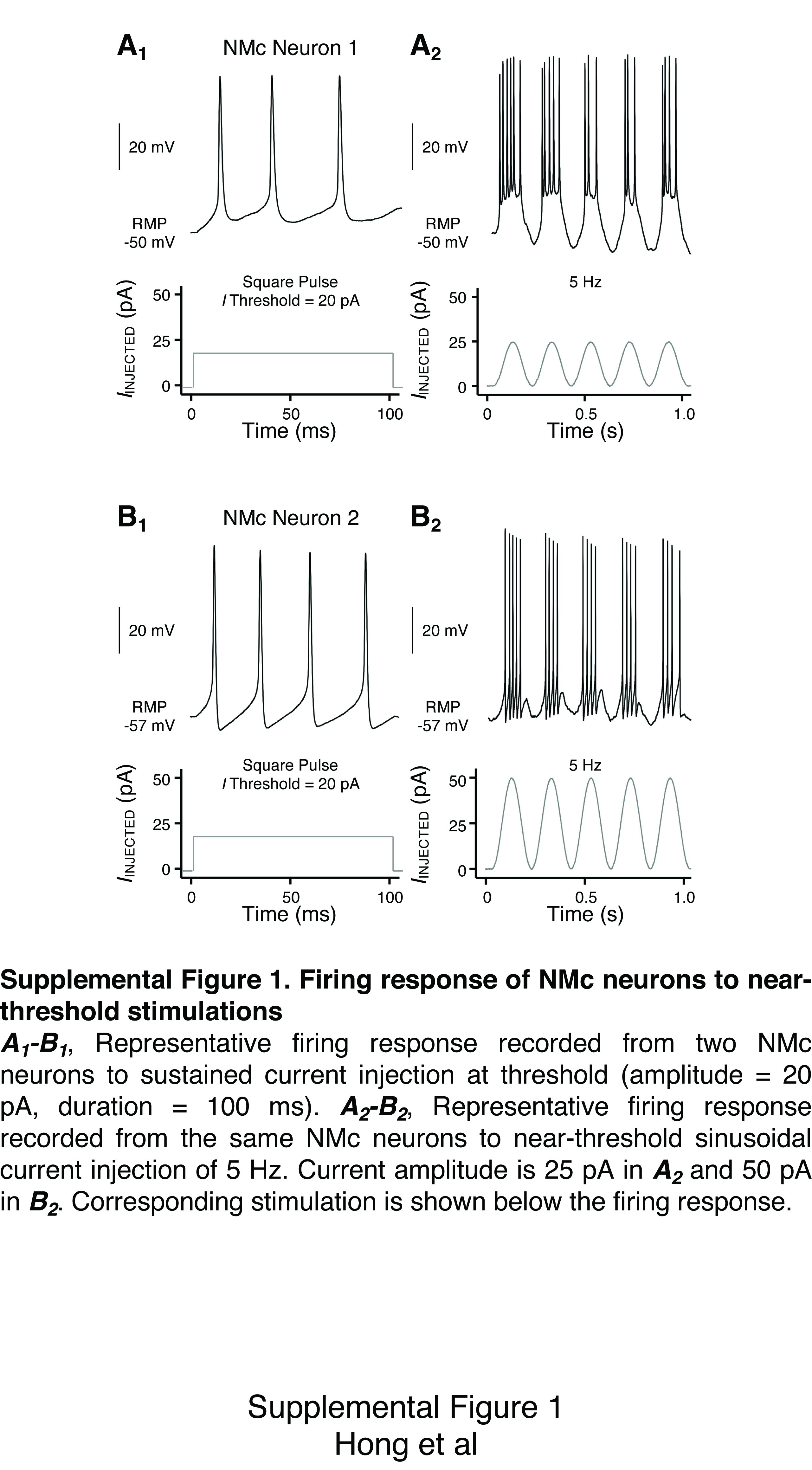

Supplement: Supplementary file 1 [file Image_1.TIF]
